# Synergistic Cascade Strategy Based on Modifying Tumor Microenvironment for Enhanced Breast Cancer Therapy

**DOI:** 10.3389/fphar.2021.750847

**Published:** 2021-11-15

**Authors:** Huan Zhang, Jinshun Xu, Binyang Gao, Hong Wang, Jianbo Huang, Jie Zhou, Rui Yang, Feng Yan, Yulan Peng

**Affiliations:** ^1^ Department of Ultrasound, West China Hospital, Sichuan University, Chengdu, China; ^2^ Laboratory of Ultrasound Imaging Drug, West China Hospital, Sichuan University, Chengdu, China

**Keywords:** breast cancer, tumor microenvironment, drug delivery, hyperthermia, nanoparticles

## Abstract

**Background:** Triple-negative breast cancer (TNBC) is the most aggressive subtype of breast cancer with very few treatment options. Although tumor-targeted nanomedicines hold great promise for the treatment of TNBC, the tumor microenvironment (TME) continues to be a major cause of failure in nanotherapy and immunotherapy. To overcome this barrier, we designed a new synergistic cascade strategy (SCS) that uses mild hyperthermia and smart drug delivery system (SDDS) to alter TME resistance in order to improve drug delivery and therapeutic efficacy of TNBC.

**Methods:** Mild hyperthermia was produced by microwave (MW) irradiation. SDDS were formulated with thermosensitive polymer-lipid nanoparticles (HA-BNPs@Ptx), composed of polymer PLGA, phospholipid DPPC, hyaluronic acid (HA, a differentiation-44-targeted molecule, also known as CD44), 1-butyl-3-methylimidazolium-L-lactate (BML, a MW sensitizer), and paclitaxel (Ptx, chemotherapy drug). 4T1 breast tumor-bearing mice were treated with two-step MW combined with HA-BNPs@Ptx. Tumors in mice were pretreated with first MW irradiation prior to nanoparticle injection to modify and promote TME and promoting nanoparticle uptake and retention. The second MW irradiation was performed on the tumor 24 h after the injection of HA-BNPs@Ptx to produce a synergistic cascade effect through activating BML, thus, enhancing a hyperthermia effect, and instantly releasing Ptx at the tumor site.

**Results:** Multifunctional CD44-targeted nanoparticles HA-BNPs@Ptx were successfully prepared and validated *in vitro*. After the first MW irradiation of tumors in mice, the intratumoral perfusion increased by two times, and the nanoparticle uptake was augmented by seven times. With the second MW irradiation, remarkable antitumor effects were obtained with the inhibition rate up to 88%. In addition, immunohistochemical analysis showed that SCS therapy could not only promote tumor cell apoptosis but also significantly reduce lung metastasis.

**Conclusion:** The SCS using mild hyperthermia combined with SDDS can significantly improve the efficacy of TNBC treatment in mice by modifying TME and hyperthermia-mediated EPR effects.

## Introduction

Breast cancer is the most common cancer and one of the leading causes of cancer-related death in women worldwide (Tajbakhsh et al., 2018). Despite the early detection and intervention, metastatic breast cancers remain largely incurable, especially triple-negative breast cancer (TNBC) (Mu et al., 2017; Thakur and Kutty, 2019). TNBC is an aggressive subtype of breast cancer and accounts up to 10%–20% of all breast cancer cases (Ding et al., 2021). Due to the lack of specific targets and high probability of metastasis, currently available treatment options are very limited (Hu et al., 2020). Metastatic breast cancer is characterized by a unique tumor microenvironment (TME), which differs from other subtypes. The components of TME, including transformed extracellular matrix (ECM), soluble factors, immune suppressive cells, epigenetic modifications, and re-programmed fibroblasts, together hamper antitumor response and help in the progression and metastasis of TNBC (Junttila and de Sauvage, 2013; Deepak et al., 2020). Another barrier to breast cancer is its high heterogeneity, which complicates treatment (Pashayan et al., 2020; Wang et al., 2020). For example, a small piece of tumor tissue obtained by biopsy does not necessarily represent all the tumor components (Januškevičienė and Petrikaitė, 2019). In addition, high interstitial fluid pressure (IFP) generated by TME also severely limits drug delivery to tumor cells, especially in immunotherapy and nanotherapy (Yang and Gao, 2017). High IFP in TME may compress blood vessels, resulting in reduced intratumoral blood flow and nanodrug delivery (Griffon-Etienne et al., 1999; Nia et al., 2020). Since the TME is involved in the proliferation, angiogenesis, apoptosis inhibition, immune system suppression, and drug resistance of metastatic breast cancer, it becomes recently an important target of TNBC therapy (Nienhuis et al., 2015; Lang et al., 2019; Feng et al., 2020).

Hyperthermia combined with nanoparticles loaded with chemotherapeutic agents holds great promise for the treatment of cancer (Dunne et al., 2020). However, the efficacy in solid cancer treatment has not been clinically proven. For example, numerous clinical trials have been conducted since 2006 using ThermoDox, doxorubicin-loaded low temperature-sensitive liposomes (LTSLs), to treat hepatic, colorectal, prostate, and breast cancer (May and Li, 2013). Hyperthermia can be achieved using different heating techniques, such as radiofrequency (RF), focused ultrasound (FUS), and microwave (MW) (Markezana et al., 2020). A phase I study (TARDOX) recently showed that the combined treatment of LTSLs and non-invasive FUS hyperthermia seems to be clinically feasible, safe, and able to enhance intratumoral drug delivery (Lyon et al., 2018). Although an increased intratumoral drug delivery has been demonstrated in a preclinical study, most of phase II and phase III trials of ThermoDox failed to demonstrate the benefit of the combined treatment over chemo- or thermal therapy alone (Tak et al., 2018; De Maar et al., 2020). Therefore, the preclinical research and clinical translation of thermosensitive nanomedicines are still facing huge demands and challenges (Dou et al., 2017; Nardecchia et al., 2019).

Hyperthermia induced by MW is a promising adjuvant therapy, which can be used to induce the apoptosis of tumor cells and destroy ECM in TME (Paulides et al., 2020). Several techniques have been used to generate local heating for tumor repression, including near-infrared photothermal therapy, magnetic thermal therapy, RF thermal therapy, ultrasonic hyperthermia, and microwave thermal therapy. The MW thermal therapy of tumors has attracted much interest recently, due to the maneuverability, faster heat generation, depth of penetration in tissues, and perfect ability of killing tumor cells (Dunne et al., 2020; Paulides et al., 2020). MW can penetrate deeply into most of solid tumors in patients, does not produce drug resistance of TME, and has no systemic side effects (Wu et al., 2019). Mild MW hyperthermia can also induce apoptosis of tumor cells at 39°–45°C (Qi et al., 2019; Dunne et al., 2020). However, there are very few reports using MW hyperthermia combined with Ptx-loaded nanoparticles to treat metastatic breast cancers. Therefore, the need for combination therapy to overcome the resistance of breast tumors and improve the efficacy of current treatment is very clear.

**FIGURE 1:**
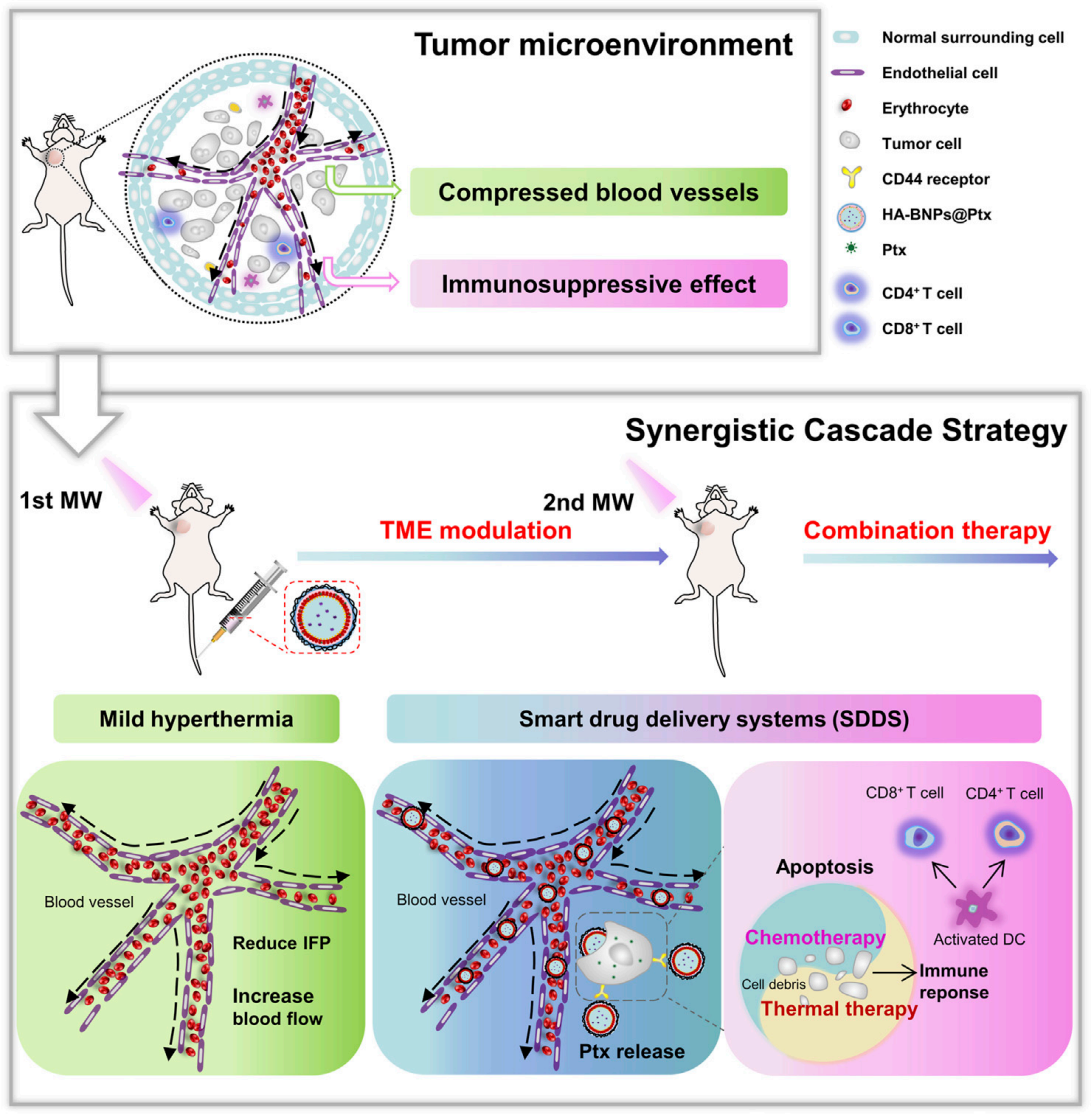
Illustration of tumor microenvironment and treatment process with synergistic cascade strategy.

In the present study, we proposed a new approach to enhance the bioavailability of nanodrugs and the efficacy of hyperthermia to treat triple negative breast cancer called “Synergistic Cascade Strategy” (SCS). The strategy consists of using local MW hyperthermia combined with smart drug delivery system (SDDS) to treat TNBC in a 4T1 breast cancer-bearing mice model. SDDS was formulated with polymer-lipid nanoparticles (HA-BNPs@Ptx), composed of polymer PLGA, phospholipid DPPC, hyaluronic acid (HA, targeting differentiation-44, also known as CD44), 1-butyl-3-methylimidazolium-L-lactate (BML, a MW sensitizer), and paclitaxel (Ptx, chemotherapy drug). Our previous work (Xu et al., 2019a) showed that MW-responsive nanoplatform made of lipid nanoparticles, containing DPPC and BML, could efficiently deliver doxorubicin and inhibit hepatocellular carcinoma progression with distant lung metastasis. The rationale of SCS is the following: (Tajbakhsh et al., 2018) performing the first MW on tumor to induce mild hyperthermia (∼43°C), in order to reduce IFP in TME and increase intratumoral blood flow. The mild hyperthermia also allows “permeabilizing” tumoral vessels and alter the tumor surrounding matrix, thus, promoting the extravagation and penetration of nanoparticles to reach tumor cells (Thakur and Kutty, 2019); injecting long circulating target PLGA-DPPC nanoparticles immediately following the first MW exposure to allow the maximal accumulation of nanoparticles at tumor sites (Mu et al., 2017); and after nanoparticle accumulation, performing second MW exposure to activate the sensitizer BML, thus, increasing rapidly the temperature inside the tumor and instantly release Ptx from nanoparticles to induce tumor cell apoptosis and destroy the ECM in TME, though there are synergistic cascade effects of hyperthermia and chemotherapy agents. Figure 1 illustrates the scenario of the above approach.

## Materials and methods

### Materials

NH_2_-PLGA-NH_2_ (lactide:glycolide, 50/50, MW: 8 kDa) was obtained from Xi’an Rui-Xi Biological Technology Co. Ltd. (Xi’an, Shaan Xi, China). 1-Butyl-3-methylimidazolium-L-lactate (BML, MW: 228.29 Da) was provided by Cheng Jie Chemical Co. Ltd. (Shanghai, China). Hyaluronic acid (HA, MW: 3.8 kDa) was purchased from Dalian Meilun Biotechnology Co. Ltd. (Dalian, Liao Ning, China). Paclitaxel (Ptx, MW: 853.93 Da) was purchased from Shanghai Aladdin Biotechnology Co. Ltd. (Shanghai, China). 1,2-Dipalmitoyl-sn-glycerol-3-phosphocholine (DPPC, MW: 734.1 Da), polyvinyl alcohol (PVA, MW: 30–70 kDa), 2-(N-morpholino) ethane sulfonic acid (MES), N-hydroxysuccinimide (NHS), and 1-ethyl-3-(3-dimethylaminopropyl) carbodiimide (EDC) were obtained from Sigma-Aldrich Corporation (St. Louis, MO, USA). All reagents used in this work were of analytical grade without further purification.

### Preparation of HA-BNPs@Ptx

The double emulsification method was used to synthesize Ptx and BML-loaded DPPC-PLGA hybrid nanoparticles (BNPs@Ptx) following a previous report (Xu et al., 2019b). Briefly, 10 mg of DPPC, 20 mg of NH_2_-PLGA-NH_2_, and 2 mg of Ptx were thoroughly dissolved in 2 ml of dichloromethane (DCM). BML, 400 µl (0.5 mg/ml, in deionized water), was mixed in the solution. The Ptx and BML solution was emulsified in an ice bath (2 min, with a 5-s on–off duty cycle) using an ultrasonic oscillation instrument (SCIENTZ-IID, Ningbo Scientz Biotechnology Co., Ltd., Ningbo, Zhejiang, China) at 12% power (24 kHz, 600 W). PVA, 8 ml (2% w/w, in water) solution, was further added to the emulsion and emulsified as before. DCM was then evaporated in a fume hood for 3 h under magnetic stirring. Finally, BNPs@Ptx was collected by centrifugation at 10,000 × *g* for 10 min and washed twice with double distilled water. Nanoparticles targeting CD44 were prepared by covalently coupling hyaluronic acid to BNPs@Ptx via amino groups on PLGA (Alam et al., 2017). Briefly, 2 mg of HA was dissolved in 195 mg of MES buffer (pH = 5.5). EDC (27 mg) and 8.6 mg of NHS were then added to the HA solution. Subsequently, the mixture was incubated with 1 ml of BNPs@Ptx suspension under continuous stirring for 24 h on ice bath. HA-BNPs@Ptx was collected and purified by centrifugation and washed with double distilled water two times.

### Characterization of HA-BNPs@Ptx

#### Morphology and physicochemical properties of HA-BNPs@Ptx

Transmission electron microscopy (TEM, Tecnai G2 F30, FEI Co., Ltd., Hillsboro, OR, USA) and scanning electron microscope (SEM, SU8020, Hitachi, Ltd., Tokyo, Japan) were used to observe the morphology of the nanoparticles. The size distribution, polydispersity index (PDI), and zeta potential were obtained using dynamic light scattering (DLS, Brookhaven Omni, Brookhaven Instruments Inc., Holtsville, NY, USA). The Fourier transform infrared spectrometer (FTIR, Nicolet iS10, Thermo Fisher Scientific Co., Ltd., MA, USA) was used to record the spectra of HA, BNPs@Ptx, and HA-BNPs@Ptx in the wavelength range of 500–4,000 cm^−1^. The drug loading (DL) and encapsulation efficiency (EE) of Ptx were quantified using a standard calibration curve measured at 229 nm on a visible ultraviolet spectrophotometer (L5S, INESA Analytical Instrument Co., Ltd., Shanghai, China, Supplementary Material, Methods 1.1 for details).

#### Microwave-induced thermal effect and paclitaxel release of HA-BNPs@Ptx

To evaluate the thermal effect of MW irradiation with sensitizer BML, 1 ml of HA-BNPs@Ptx or HA-NPs@Ptx suspensions was added to a 24-well plate and exposed to MW energy instrument (WZY-1, Beijing Muheyu Electronics Co., Ltd, Beijing, China). The suspensions were irradiated for 4 min at different MW power (0.4, 0.8, 1.2, and 1.6 W cm^−2^, 450 MHz). The temperature change in the HA-BNPs@Ptx suspension was recorded every minute using an infrared thermal mapping instrument (FLUKE 572-2, Hawk-IR International, Inc., Everett, WA, USA). Ptx release kinetics upon MW heating was evaluated by using visible ultraviolet spectrophotometer.

### *In vitro* biocompatibility and targeting ability of HA-BNPs@Ptx

#### Cytotoxicity assay

Mouse breast cancer cell line 4T1, mouse macrophage cell line J774, and human umbilical vein endothelial cells (HUVECs) were purchased from Cellcook (Guangzhou Cellcook Cell Biotechnology, Ltd., Guangzhou, Guangdong, China) and cultured according to the instructions of the supplier. 4T1 cells and HUVECs were seeded into a 96-well plate (1 × 10^4^ cells per well) and incubated overnight. Five duplicate holes were set in each group. Subsequently, 100 µl of HA-BNPs@Ptx at Ptx concentrations (2, 5, 10, 20, and 50 µg/ml) was introduced to each group. The cytotoxicity of HA-BNPs@Ptx was examined by CCK-8 viability assay (Boster Biological Technology Co., Ltd., CA, USA). The optical density (OD) was measured at 450 nm by a Varioskan Flash microplate reader (Synergy Mx, BioTek Instruments, Inc., Winooski, VT, USA). The cytotoxicity treated with HA-NPs (without drug loaded NPs) was evaluated using the same method.

#### Hemolytic activity

Two percent suspension of chicken red blood cells was used to determine the hemolytic activity of HA-BNPs@Ptx. Phosphate buffer saline (PBS; 100 µl), double distilled water, or HA-BNPs@Ptx suspensions at Ptx concentrations of 2, 5, 10, 20, and 50 µg/ml were added to erythrocyte suspensions (100 µl). PBS was used as the negative control group, and double distilled water was used as the positive control group. All suspensions were incubated at 37°C for 1 h and centrifuged at 800 × *g* for 10 min. The OD was measured at 550 nm using the Varioskan Flash microplate reader.

#### CD44 receptor mediated nanoparticle targeting

4T1 cells were used to verify CD44 targeting ability of nanoparticles. The CD44 expression level of 4T1 cells was evaluated by an inverted fluorescence microscope (Supplementary Material, Methods 1.2 for details). The targeting test was performed using BNPs@Ptx, HA-BNPs@Ptx, and HA-BNPs@Ptx with the cells in which CD44 receptor was pre-saturated by an excess amount of free HA (Zhang et al., 2020). To obtain stained nanoparticles, 1 ml of BNPs@Ptx or HA-BNPs@Ptx suspensions were stained with 10 µl of DiO (1:100). After overnight culture, 4T1 cells were co-incubated with 100 µl of BNPs@Ptx or HA-BNPs@Ptx for 6 h. The targeting ability was visualized by the inverted fluorescence microscope (AX10 imager A2/AX10 cam HRC, Carl Zeiss, Co., Ltd., Jena, Germany). Flow cytometry (FCM, Cyto Flex, Beckman Coulter, Inc., CA, USA) was used to further evaluate the ability of HA-BNPs@Ptx to target 4T1 cells at different time intervals (30 min, 1, 3, 6, and 24 h).

### *In vitro* cellular uptake and intracellular tracking

To examine cellular uptake and intracellular trafficking, BNPs@Ptx and HA-BNPs@Ptx were labeled with a red fluorescence probe Dil. Endocytosis/phagocytosis experiments were performed in 4T1 cells and J774 cells (murine macrophages), respectively. 4T1 cells per well (1 × 10^4^) were seeded in confocal dishes. After 24 h, 100 µl of DiI-BNPs@Ptx and DiI-HA-BNPs@Ptx was added to co-incubate with 4T1 cells for 1, 3, and 6 h, respectively. At each time point, 20 µl of Lyso Tracker Green was added to each confocal dish to stain endo/lysosomes. One and a half hours after incubation with Lyso Tracker Green, all cells were fixed with 4% paraformaldehyde for 10 min. 4,6-Diamidino-2-phenylindole (DAPI, blue, 10 µl) was finally added in the fixed cells to stain the nuclei. Confocal fluorescence images of the fixed cells were obtained by a laser scanning confocal microscope (LSCM, A1R + MP, Nikon Co., Tokyo, Japan).

J774 cells were treated in the same way as 4T1 cells, except that 5 × 10^4^ cells and 10 µl of PMA (100 ng/ml) were added to each dish and incubated for 48 h.

### *In vitro* synergistic anti-tumor ability

Synergistic anti-cancer activity of thermal chemotherapy was evaluated with 4T1 cells *in vitro*. The cells were seeded into a six-well plate (7.5 × 10^5^ cells per well) for 24 h and then incubated with 1) PBS, 2) HA-BNPs@Ptx, 3) MW, 4) BNPs@Ptx + MW, an5) HA-BNPs@Pd tx + MW for 6 h. In order to optimize the thermal effects of nanoparticles, HA-BNPs@Ptx/4T1 cells were further exposed to MW irradiation at different power (0.4, 0.8, 1.2, and 1.6 W cm^−2^) for 1, 2, 3, and 4 min, respectively (see Supplementary Material, Methods 1.3 for details). After incubation, the cells of the last three groups were exposed to MW irradiation (0.8 W cm^−2^, 4 min). The anti-cancer activity of each group was qualitatively examined by using calcein-AM/propidium iodide (PI) double stain kit (Beyotime Biotechnology® Inc., Suzhou, Jiangsu, China). After LIVE/DEAD staining of the cells, all cell samples were imaged under an inverted fluorescence microscope.

To quantitatively assess the synergistic chemo-thermal therapy, 4T1 cells were stained by Annexin V-(FITC)/PI apoptosis detection kit (4A Biotech Co., Ltd., Peking, China) and analyzed by flow cytometer.

### *In vivo* triple-negative breast cancer models, biodistribution, and targeting ability

BALB/c mice (female, 18–20 g) were provided by Dashuo Biological Technology (Chengdu, Sichuan, China). All processes were in accordance with the Chinese Society of Laboratory Animals on animal welfare and approved by the Animal Use and Care Management Advisory Committee of West China Hospital of Sichuan University (Approval No. 2017014A). 4T1 cells (1 × 10^6^/wells) were implanted into the second axillary mammary fat pad on the right side. When the volume of tumor reached about 100 mm^3^, 4T1 breast tumor-bearing mice were randomly assigned into four groups (six mice per group): 1) DiI-BNPs@Ptx, 2) DiI-HA-BNPs@Ptx, 3) MW + DiI-BNPs@Ptx, and 4) MW + DiI-HA-BNPs@Ptx to compare the effect among non-targeted (EPR), CD44-targeted (active targeting), and MW irradiation (physical targeting). MW + DiI-BNPs@Ptx and MW + DiI-HA-BNPs@Ptx were treated with mild hyperthermia (4-min MW exposure at 0.8 W cm^−2^). Then nanoparticles (0.5 μl/g, each microliter of nanoparticle suspension contained 0.434 μg of Ptx and 1.45 μg of nanoparticles) were injected into 4T1 breast tumor-bearing mice via tail vein. *In vivo* fluorescence images were collected before and after injection of nanoparticles at 1, 3, 6, and 24 h using the IVIS Spectrum system (Lumina XR, Caliper Life Sciences, Boston, MA, USA). To examine the biodistribution of nanoparticles, the main organs (heart, liver, spleen, lungs, and kidneys) and tumors were isolated from mice to perform *ex vivo* imaging using the same IVIS Spectrum.

Blood chemistry analysis of BALB/c mice was made 7 days after intravenous injection with HA-BNPs@Ptx (Supplementary Material, Methods 1.4 for details).

### *In vivo* mild hyperthermia—the first microwave irradiation

The first MW irradiation aimed to generate mild hyperthermia to alter the TME to increase the uptake of nanoparticles. Six mice in each group received mild hyperthermia at the tumor site with a MW power of 0.8 W cm^−2^. The temperature of tumor was monitored in real time using an infrared thermal mapping instrument. After mild hyperthermia, DiI-HA-BNPs@Ptx (0.5 μl/g, each microliter of nanoparticle suspension contained 1.45 μg of nanoparticles) was immediately injected into 4T1 breast tumor-bearing mice via tail vein. The mice were euthanized, and the tumor tissue was removed after 24 h. The retention of nanoparticles (red fluorescence) in tumor tissues was revealed using a pathological section scanner Pannoramic DESK (P-MIDI-P250, 3D HISTECH, Budapest, Hungary).

To further explore changes in TME, intratumoral perfusion and micro-vessel density of tumor were evaluated using contrast-enhanced ultrasound imaging (CEUS) and CD31 immunohistochemical analysis. Microbubbles (0.2 ml/kg, SonoVueTM, Bracco, Italy) were injected *via* tail vein 24 h after the first MW irradiation. CEUS was performed using an ultrasound scanner (iU22, Koninklijke Philips N.V., Eindhoven, Netherlands) with a 12- to 5-MHz transducer. Subsequently, mice were euthanized, and tumors were sectioned and stained with CD31 (dilution 1:50, Wuhan Service Bio Co., Ltd., Wuhan, Hubei, China) to evaluate the micro-vessel density. The pathological sections were imaged using a fluorescence microscope.

### *In vivo* anti-tumor efficiency—the second microwave irradiation

The second MW irradiation was dedicated to activate the sensitizer BML and to release the chemotherapy agent Ptx in the nanoparticles. Thirty 4T1 breast tumor-bearing mice were divided into five groups: PBS (control group, G1) (Tajbakhsh et al., 2018), first MW + HA-BNPs@Ptx (G2) (Thakur and Kutty, 2019), first MW + HA-BNPs + second MW (G3) (Mu et al., 2017), HA-BNPs@Ptx + second MW (conventional SDDS, G4) (Ding et al., 2021), and first MW + HA-BNPs@Ptx + second MW (G5) (Hu et al., 2020). After treatment with mild hyperthermia (4-min MW exposure at 0.8 W cm^−2^, G2, G3, and G5), PBS, HA-BNPs, or HA-BNPs@Ptx (0.5 μl/g, each microliter of nanoparticle suspension contained 0.434 μg of Ptx) was immediately injected into 4T1 breast tumor-bearing mice intravenously. Twenty-four hours after injection, the mice received a second MW irradiation (0.8 W cm^−2^) for 4 min. The tumor temperature was monitored in real time by infrared thermal mapping instrument. The body weight and tumor volumes were recorded every 3 days. The tumor suppression rate (TSR) was calculated using the following formula:

TSR(%)=(Vc-Vx)/Vc×100%

where Vc is the volume of the PBS group, and Vx is the volume of the treatment group.

On day 18 after treatment, tumors and main organs were excised and fixed overnight in 10% buffered formalin. The tumors were sectioned and stained with H&E, Ki-67 antibody, and TdT-mediated dUTP nick-end labeling (TUNEL) staining. The main organs were sectioned and stained with H&E. The expression level of CD3^+^, CD4^+^, and CD8^+^ of immune cells in tumor tissues were evaluated by immunofluorescence. The slides of tumor tissues were incubated with Anti-CD3 Rabbit pAb (P22646,1:700), Anti-CD4 Rabbit pAb (P06332 1:800), and Anti-CD8 Rabbit mAb (P10966, 1:500), following the standard procedure of Wuhan Service Bio Co., Ltd. (Wuhan, Hubei, China). The nucleus was labeled with DAPI. The images were obtained by using the pathological section scanner Pannoramic DESK.

In addition, peripheral blood was collected from treated mice before euthanasia to analyze the percentages of tumor antigen-specific CD3^+^, CD4^+^, and CD8^+^ T cells. Serum cells were incubated with 3 µl of FITC anti-mouse CD3 antibody, 5 µl of PE anti-mouse CD4, and 10 µl of APC anti-mouse CD8 (4A Biotech Co., Ltd., Peking, China) for 1 h at room temperature, respectively. Finally, the cells were resuspended in PBS and analyzed by flow cytometry and Flow Jo software.

### Statistical analysis

All data were expressed as mean ± standard deviation (SD). The statistical analysis was carried out with GraphPad Prism Version 8.0 software (GraphPad, USA). Comparisons among multiple groups were performed by one-way analysis of variance (ANOVA). Two-group comparisons were performed by Student’s *t*-test. Statistical significance was indicated by * for *p* < 0.05, ** for *p* < 0.01, and *** for *p* < 0.001.

## Results

### The characterization of HA-BNPs@Ptx

**FIGURE 2:**
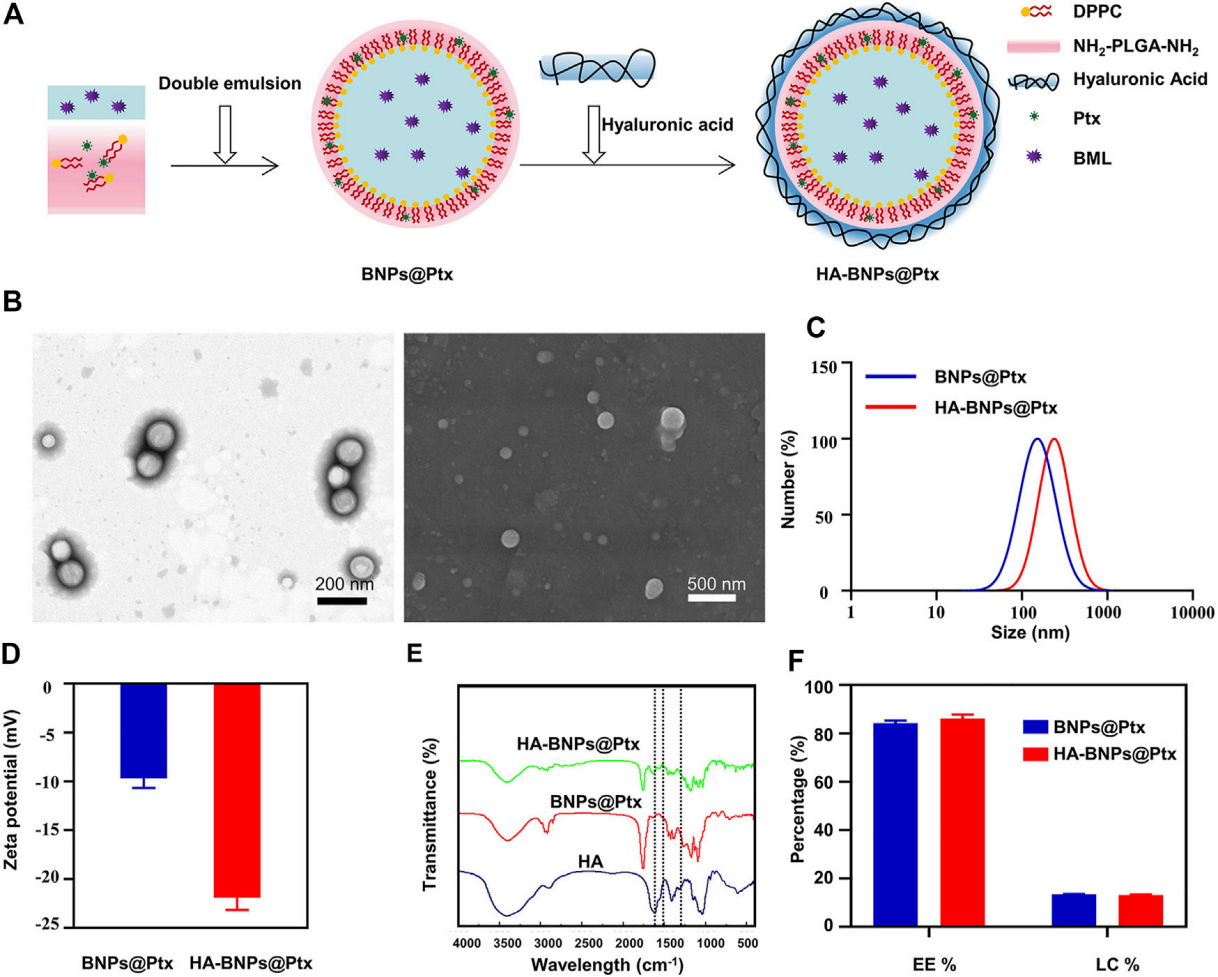
Synthesis and characterization of nanoparticles used in synergistic cascade strategy study. **(A)** BNPs@Ptx preparation and hyaluronic acid (HA) conjugation. **(B)** Transmission electron microscopy (TEM) and scanning electron microscope (SEM) image of HA-BNPs@Ptx. **(C)** Size distribution and **(D)** zeta potentials of BNPs@Ptx and HA-BNPs@Ptx determined by dynamic light scattering (DLS) (*n* = 3). **(E)** Fourier transform infrared spectrometer (FTIR) spectrums of HA, BNPs@Ptx, and HA-BNPs@Ptx (amide bond: 1,656, 1,546, and 1,334 cm^−1^). **(F)** Encapsulation efficiency (EE) and loading capacity (LC) of Ptx in HA-BNPs@Ptx and BNPs@Ptx determined by spectrophotometer (*n* = 3).

**Figure 2A** illustrates the preparation procedure of nanoparticles HA-BNPs@Ptx. TEM and SEM images showed a core–shell structure of the nanoparticles with a mean diameter of about 150 nm, spherical shaped, and with a smooth surface (Figure 2B). DLS revealed that HA-BNPs@Ptx was 203.30 ± 7.51 nm and relatively homogenous (PDI: 0.172, Figure 2C). HA-BNPs@Ptx was negatively charged with zeta potential of −22 mV (Figure 2D) and stable at neutral pH (Supplementary Figures S1A,B). The vibration absorption peaks of amide bonds at 1,656, 1,546, and 1,334 cm^−1^ in FTIR spectrums confirmed the successful conjugation of HA to BNPs@Ptx nanoparticles (Figure 2E). Ptx loading capacity of HA-BNPs@Ptx determined by spectrophotometer was 13.17 wt.%, corresponding to encapsulation efficiency of 85.62 wt.% (Figure 2F).

### *In vitro* microwave-induced 1-butyl-3-methylimidazolium-L-lactate thermal effect and drug release

**FIGURE 3:**
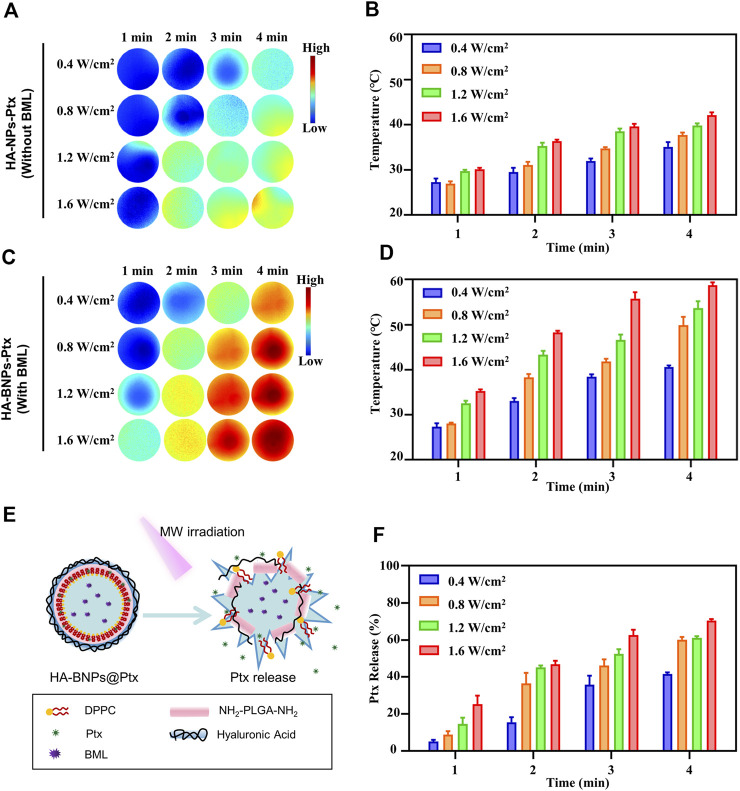
*In vitro* evaluation of microwave (MW) thermal effect and paclitaxel (Ptx) release. **(A, B)** IR thermal imaging and temperature histogram of HA-NPs@Ptx [without thermosensitive agent 1-butyl-3-methylimidazolium-L-lactate (BML)] at different MW power and exposure time. **(C, D)** IR thermal imaging and temperature histogram of HA-BNPs@Ptx (with thermosensitive agent BML). **(E)** Schematic illustration of HA-BNPs@Ptx BML activation and Ptx release under MW irradiation. **(F)** *In vitro* release of Ptx from HA-BNPs@Ptx exposed at different MW power and irradiation time. The data are presented as mean ± SD (*n* = 3).

In order to optimize the MW thermal effect and Ptx release, nanoparticle suspensions (HA-NPs@Ptx and HA-BNPs@Ptx) were exposed at different MW power and time. The infrared thermal imaging pictures showed that under the same irradiation, the heating effect of HA-NPs@Ptx without BML (Figures 3A,B) was significantly lower than that of HA-BNPs@Ptx with the sensitizer (Figures 3C,D). For HA-BNPs@Ptx, the temperature of the suspension increased rapidly from room temperature to 35.2°C (0.4 W cm^−2^) and reached nearly 60°C (1.6 W cm^−2^) under 4-min MW exposure. Due to the lack of BML, the temperature of HA-NPs@Ptx increased only to 30.4°C at 0.4 W cm^−2^ and 41.8°C at 1.6 W cm^−2^. These results suggest that HA-BNPs@Ptx has a greater potential to trigger Ptx release in combination with thermal chemotherapy.

Figures 3E,F show the release of Ptx from HA-BNPs@Ptx upon MW irradiation. The Ptx solution standard curve was established according to previous reports to quantify Ptx (Supplementary Figure S2) (Furman et al., 2017). A maximum release of 70% was obtained at 1.6 W cm^−2^ for 4 min. At this condition, the temperature was very high (58°C, Figure 3D), and this would directly induce tumor necrosis (Seynhaeve et al., 2020; Pan et al., 2021). In order to avoid excessive heat and demonstrate our synergistic cascade strategy, a lower MW power of 0.8 W cm^−2^ was chosen for subsequent *in vitro* and *in vivo* studies.

### *In vitro* targeting ability, cellular uptake, and anti-tumor effect

Hyaluronic acid, as a CD44-specific ligand, is widely used in various targeted drug delivery systems for TNBC (Zhang et al., 2020; Sun et al., 2019). The expression of CD44 on 4T1 cells was verified (Supplementary Figure S3A), and the specific targeting ability of nanoparticles to 4T1 cells was compared between HA-BNPs@Ptx and BNPs@Ptx (Supplementary Figure S3B). Supplementary Figure S3A reveals that the nanoparticles contain HA bonded fairly to 4T1 cells, and the binding was specific because the binding could be inhibited by adding the free hyaluronic acid to 4T1 cells before the incubation with the nanoparticles (Cerqueira et al., 2017). Non-specific binding could be observed with BNPs@Ptx, but the number of fluorescent nanoparticles was significantly less than that of HA-BNPs@Ptx, suggesting that the specific binding of HA and CD44 could be used to improve the cellular internalization efficiency of nanoparticles (Supplementary Figure S3B).

**FIGURE 4:**
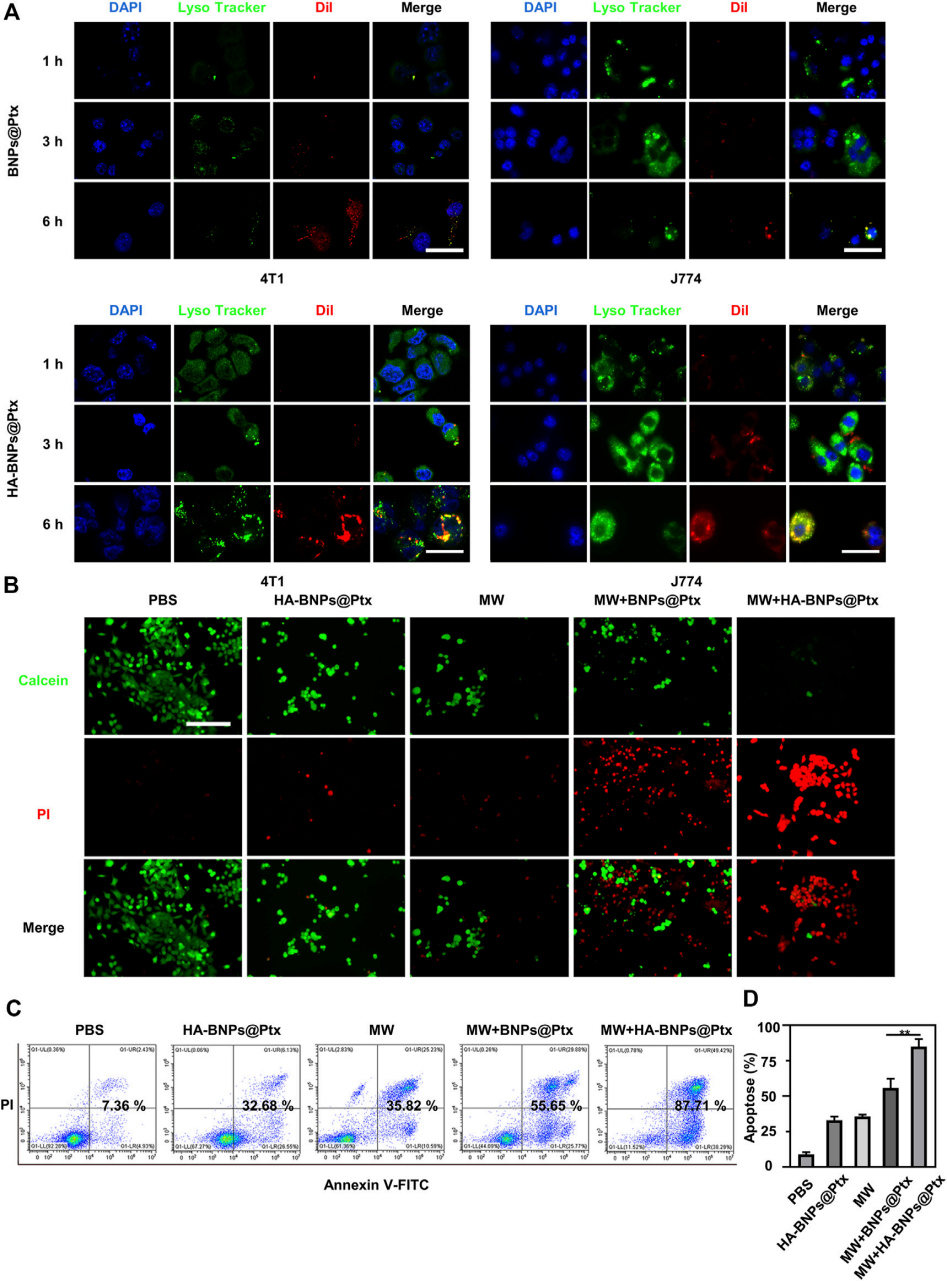
*In vitro* endocytosis and apoptosis studies. **(A)** Representative confocal imaging of 4T1 breast cancer cells and J774 macrophages incubated with BNPs@Ptx and HA-BNPs@Ptx for 1, 3, and 6 h at 37°C. The cell nuclei were stained by 4,6-diamidino-2-phenylindole (DAPI) (blue), the endo/lysosomes by Lyso Tracker Green (green), and HA-BNPs@Ptx were labeled with DiI (red). Scale bars represent 20 μm. **(B)** Fluorescence images of 4T1 cells treated with phosphate buffer saline (PBS), HA-BNPs@Ptx, MW, MW + BNPs@Ptx, or MW + HA-BNPs@Ptx. Live cells were stained by Calcein-AM (green) and dead cells by PI (red). Scale bars represent 50 μm. **(C)** After treatment, early and late apoptotic cells were determined by flow cytometry using the Annexin V-Alexa Fluor 488/Propidium Iodide Apoptosis Detection kit. **(D)** Total cell apoptosis rate was detected by flow cytometry. The data are presented as mean ± SD (*n* = 3, **p < 0.01).

The cellular uptake of HA-BNPs@Ptx was evaluated with 4T1 cells and J774 cells. The cell nuclei were stained by DAPI (blue), the endo/lysosomes were stained by LysoTracker Green (green), and the nanoparticles were labeled with DiI (red). Figure 4A shows that BNPs@Ptx and HA-BNPs@Ptx were taken up both by 4T1 and J774 cells, and stayed in endo/lysosomes. However, HA-BNPs@Ptx leads to higher fluorescent intensity compared with BNPs@Ptx. Furthermore, the uptake of HA-BNPs@Ptx by 4T1 cells was quantified using flow cytometry. A maximum uptake was observed after 6 h of incubation (Supplementary Figure S4). A lower fluorescent intensity obtained at 24 h suggests probably a partial degradation of nanoparticles in endo/lysosomes.

The synergistic antitumor effect of MW thermal chemotherapy was studied qualitatively by confocal microscope and quantitatively by flow cytometry. *In vitro* hyperthermia experiments indicate that MW irradiation alone was sufficient to induce apoptosis of 4T1 tumor cells (Supplementary Figure S5), and the effect increased with increasing exposure power and time. Figure 4B shows that the combination of MW and HA-BNPs@Ptx resulted in the most significant cell damage compared with the other tested conditions (PBS, BNPs@Ptx, MW, MW + BNPs@Ptx). The 4T1 cell apoptosis analyzed by flow cytometry presented similar results (Figures 4C,D). The apoptosis was less than 8% for the control group (PBS), 32% for HA-BNPs@Ptx alone, 35% for MW irradiation only, 55.6% for MW + BNPs@Ptx, and 87% for MW + HA-BNPs@Ptx. These results suggest that there was a synergistic effect by combining hyperthermia and Ptx-loaded nanoparticles, and also confirm the superiority of CD44-targeted nanoparticles compared with non-targeting nanoparticles.

The biocompatibility of HA-BNPs@Ptx and HA-NPs was investigated by cell counting kit8 (CCK8) assay with HUVEC and 4T1 breast cancer cells. A high cell viability (>92%) was obtained after incubation with HA-BNPs@Ptx and HA-NPs (without Ptx and BML) for 24 h for the two cell lines (Supplementary Material, Supplementary Figure S6). Supplementary Figure S7 shows that the hemolysis rate induced by HA-BNPs@Ptx is less than 5%. This confirmed that the nanoparticles had good biocompatibility and also showed good stability, with no leakage of Ptx during 24-h incubation (Nave et al., 2016).

### *In vivo* targeting ability and biodistribution of HA-BNPs@Ptx compared with BNPs@Ptx (without the absence and presence of first microwave)

**FIGURE 5:**
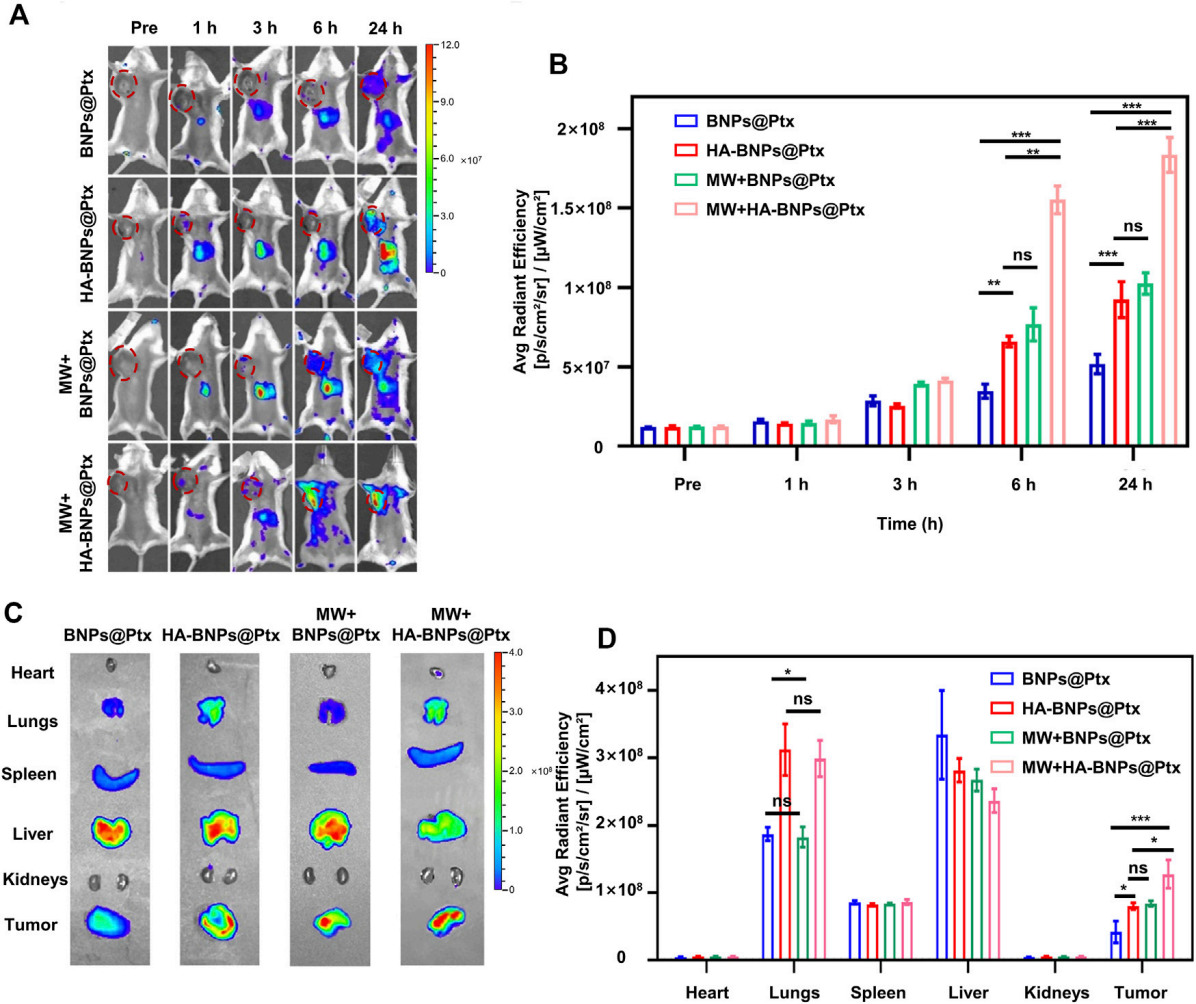
*In vivo* targeting ability and biodistribution of nanoparticles. **(A)** Representative *in vivo* fluorescence images of 4T1 breast tumor-bearing mice obtained at pre-injection, 1, 3, 6, and 24 h after intravenous injection of Dil-BNPs@Ptx and Dil-HA-BNPs@Ptx (0.5 μl/g) with or without first MW irradiation. **(B)** Average radiant intensity determined from *in vivo* fluorescence imaging for tumor-targeted (Dil-HA-BNPs@Ptx) and non-targeted (Dil-BNPs@Ptx) nanoparticles with or without first MW irradiation. **(C)** *Ex vivo* fluorescence images of main organs and tumor collected from tumor-bearing mice 24 h after injection of nanoparticles. **(D)** Average radiant intensity of main organs and tumor determined from *ex vivo* fluorescence imaging for four animal groups at 24 h after injection of nanoparticles. The data are presented as mean ± SD (*n* = 6, ns, not significant; *p < 0.05, **p < 0.01, ***p < 0.001).

To verify the targeting ability and biodistribution of HA-BNPs@Ptx, 4T1 breast tumor-bearing mice model was established in the second axillary mammary fat pad on the right side. HA-BNPs@Ptx and BNPs@Ptx were injected intravenously into 4T1 breast tumor-bearing mice, respectively. The targeting ability and biodistribution of nanoparticles were studied in real-time using a whole animal fluorescence imaging. The intensity of HA-BNPs@Ptx and BNPs@Ptx at tumor sites reached the maximum level at 24 h (Figure 5A). Compared with the non-targeted BNPs@Ptx group, the HA-BNPs@Ptx group exhibited significantly higher uptake in the tumor, almost two times higher (Figure 5B) due to the presence of HA active targeting. For untargeted BNPs@Ptx, the first MW exposure also improved significantly the EPR effect, the tumor uptake comparable with HA-BNPs@Ptx without the first MW exposure. The first MW + HA-BNPs@Ptx had the highest tumor retention rate, which is 1.7 times that of the first MW + BNPs@Ptx and HA-BNPs@Ptx, and almost four times that of BNPs@Ptx alone (EPR effect) both at 6 and 24 h after the injection of nanoparticles. Tumor tissues and major organs (heart, lung, kidney, liver, and spleen) were harvested for *ex vivo* imaging (Figure 5C). The fluorescence intensity of tumor tissues was also two times higher for the targeted nanoparticles than for non-targeted nanoparticles (Figure 5D). For other organs, comparable results were observed in the liver and spleen; a stronger fluorescence was observed in the lungs for HA-BNPs@Ptx, probably suggesting a higher CD44 expression in the lungs of the breast cancer model.

### *In vivo* mild hyperthermia (first microwave)-mediated tumor uptake of HA-BNPs@Ptx

**FIGURE 6:**
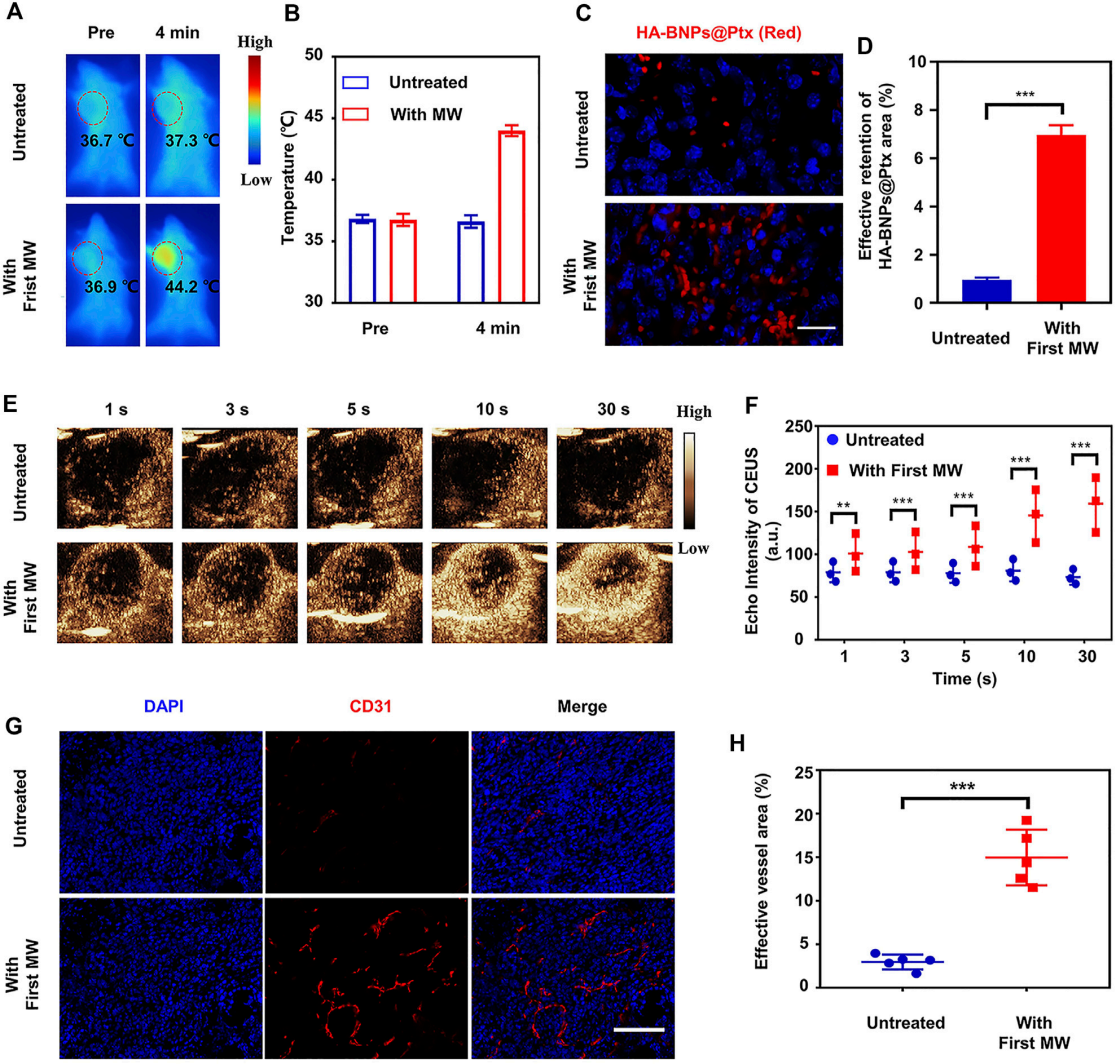
*In vivo* mild hyperthermia effects on nanoparticle uptake and retention, intratumoral flow, and tumor vasculature on 4T1 breast tumor-bearing mice. **(A)** Representative IR thermal imaging of mice with or without MW irradiation (0.8 W cm^−2^, 4 min). **(B)** Average tumor temperature before and after MW irradiation (*n* = 3). **(C)** Representative fluorescence images of uptake/retention of DiI-HA-BNPs@Ptx in tumor-bearing mice untreated and treated with MW irradiation. Images were obtained at 24 h after the treatment. **(D)** DiI-HA-BNPs@Ptx quantitative analysis of excised tumors collected from mice 24 h after treatment (*n* = 3). **(E)** *In vivo* contrast-enhanced ultrasound images obtained at different time-points after injection of microbubble contrast agent SonoVue^TM^. **(F)** Increased intratumoral perfusion was confirmed by quantifying contrast-enhanced echo intensity of MW-treated and untreated 4T1 tumors (*n* = 3). **(G)** Immunofluorescence expression of CD31 showed vascular dilation or opening within tumors, which was consistent with CEUS imaging finding. **(H)** Compared with untreated 4T1 tumor, the area of tumor blood vessels increased four times after MW irradiation. Scale bars represent 50 μm. The data are presented as mean ± SD (*n* = 5, **p < 0.01, ***p < 0.001).

The purpose of the first MW irradiation was to pretreat animals to modify TME, so as to improve the tumor uptake of nanoparticles (Yang and Gao, 2017; Hynynen, 2018). In order to understand the mechanism of the pretreatment of MW prior to the injection of HA-BNPs@Ptx, the animal was continuously monitored by infrared thermography to control the temperature change in tumors. Figures 6A,B show that the temperature of tumors was 36.7 ± 0.2°C before the first MW irradiation and increased to 43.8 ± 0.4°C after 4 min of MW irradiation (0.8 W cm^−2^). After the treatment with MW, the nanoparticles DiI-HA-BNPs@Ptx were immediately injected into mice through the tail vein (∼0.5 μl/g). Twenty-four hours after treatment, the hyperthermia effect was measured by one of the following three methods. First, red fluorescence-labeled nanoparticles that accumulated at the tumor site were detected by tissue sections (Figures 6C,D). Second, contrast-enhanced ultrasound (CEUS) imaging was performed to measure the change in intratumoral blood flow (Figures 6E,F). Third, the morphology of tumor vasculature was examined using CD31 immunofluorescent staining (Figures 6G,H).

The results showed that the retention rate of nanoparticles in the tumors irradiated by MW increased by seven times compared with the tumors not irradiated (Figures 6C,D). The echo intensity of CEUS showed that the pretreatment of tumor and its surrounding tissue by MW irradiation improved significantly intratumoral perfusion (Figures 6E,F). CD31 immunofluorescence images also showed increased tumor vascular density and/or intratumoral vasodilatation inside the tumor compared with that of tumors not irradiated (Figure 6F). Figure 6G showed that 24 h after MW exposure, the percentage of effective tumor vessels increased by a factor of about fourfold, from 3.8 ± 1.5% to 15.3 ± 4.3%. These results suggest that the pretreatment of tumors by mild hyperthermia can improve the accessibility of nanoparticles to tumors, possibly by reducing interstitial fluid pressure, dilating and increasing tumor blood vessels, and thus improving intratumoral perfusion (Chen et al., 2019).

### *In vivo* evaluation of synergistic cascade antitumor effect

**FIGURE 7:**
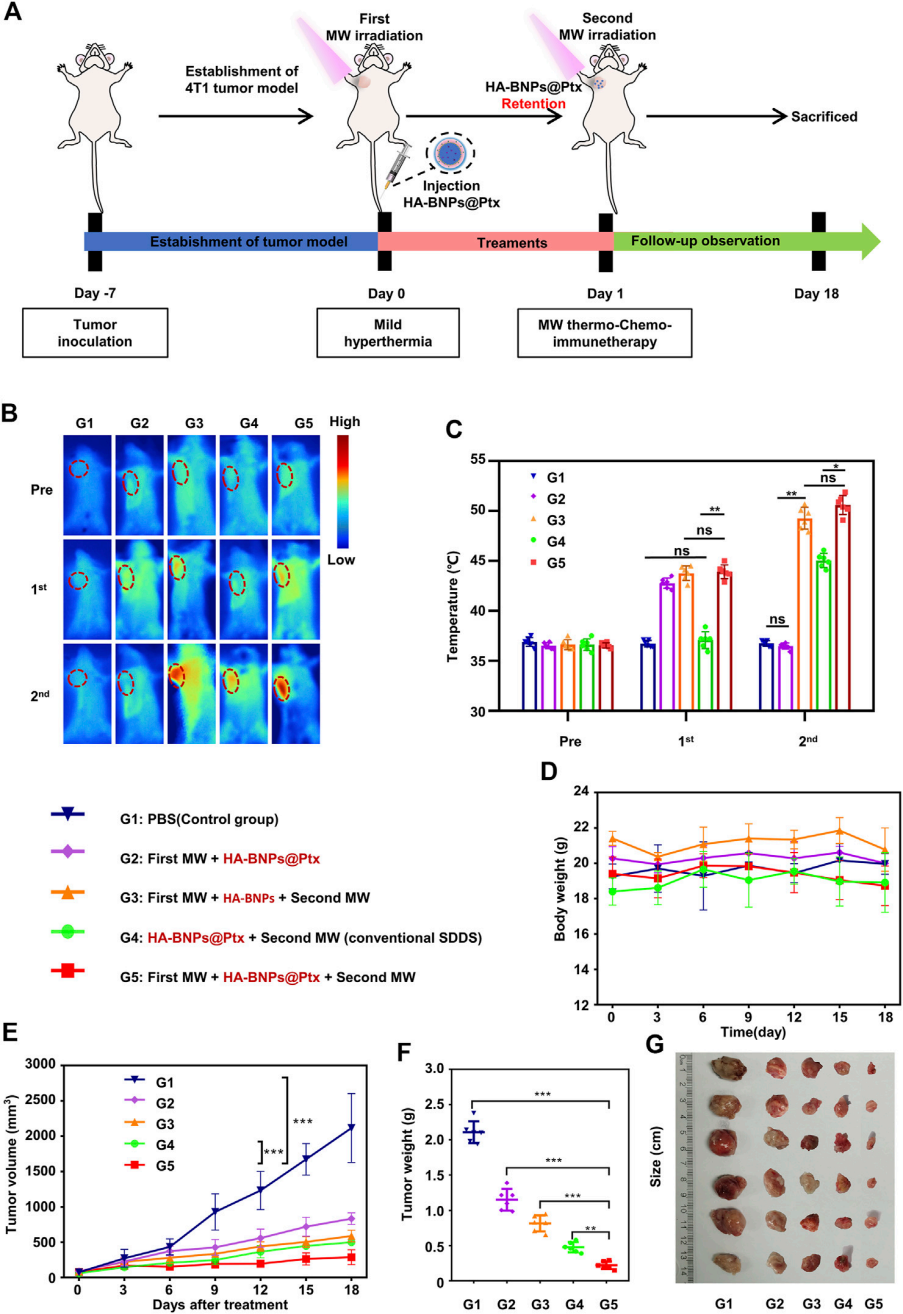
*In vivo* study of the antitumor effect of synergistic cascade strategy (double hyperthermia + nanomedicine). **(A)** Schematic illustration of the treatment process of 4T1 breast tumor-bearing mice by synergistic cascade strategy. **(B)** Representative IR thermal images of 4T1 tumor-bearing mice treated with different conditions. MW irradiation was performed at 0.8 W cm^−2^ for 4 min. **(C)** Changes in tumor temperature before and after first and second microwave exposure for different treatment mice groups (G1–G5). **(D, E)** Body weight tumor growth of mice after SCS treatment, evaluated every 3 days. **(F, G)** Changes in tumor volume and representative photograph of excised tumor of the 4T1 breast tumor-bearing mice 18 days after the treatment (G1–G5). The data are presented as mean ± SD (*n* = 6, ns, not significant; **p < 0.01, ***p < 0.001).

The synergistic cascade antitumor effect was achieved by combining two-step MW irradiations with target nanoparticles HA-BNPs@Ptx (Figure 7A). The tested conditions included group 1 (G1), PBS; group 2 (G2), first MW + HA-BNPs-Ptx; group 3 (G3), first MW + HA-BNPs + second MW; group 4 (G4), HA-BNPs-Ptx + second MW, and group 5 (G5) with first MW + HA-BNPs-Ptx + second MW. Notice the difference between G2 and G5; G2 did not receive a second MW irradiation (nanoparticles); the difference between G3 and G5 is that the former contained no Ptx (thermal therapy); the difference between G4 and G5 is that G4 did not receive the first MW irradiation (tumor pre-treatment). The group (G3, G4, and G5) that received a second MW was irradiated under the same conditions as the first one (0.8 W cm^−2^ for 4 min), and the tumor temperature was monitored in real time by an infrared thermal mapping instrument. The exposed temperature of G3 and G5 under second MW irradiation were all up to 51°C (Supplementary Material, Figures 7B,C). G4 reached to approximately 45°C. This indicates that BML encapsulated in HA-BNPs-Ptx provided supplement heating.

Figure 7D shows that throughout the treatment period, there was no significant change in animal body weight in all groups, indicating that all treatment groups were well tolerated. Compared with the other groups, the tumor volume of the G1 group increased rapidly, up to about 2,000 mm^3^ (Figures 7E–G). The antitumor effect of the G5 group was the best, with the inhibition rate of tumor growth up to 88%. G2 and G3 inhibited tumor growth by 58.2% and 67.5%, respectively. G4 also has a good antitumor effect; the inhibition rate of tumor growth was about 72.5%.

**FIGURE 8:**
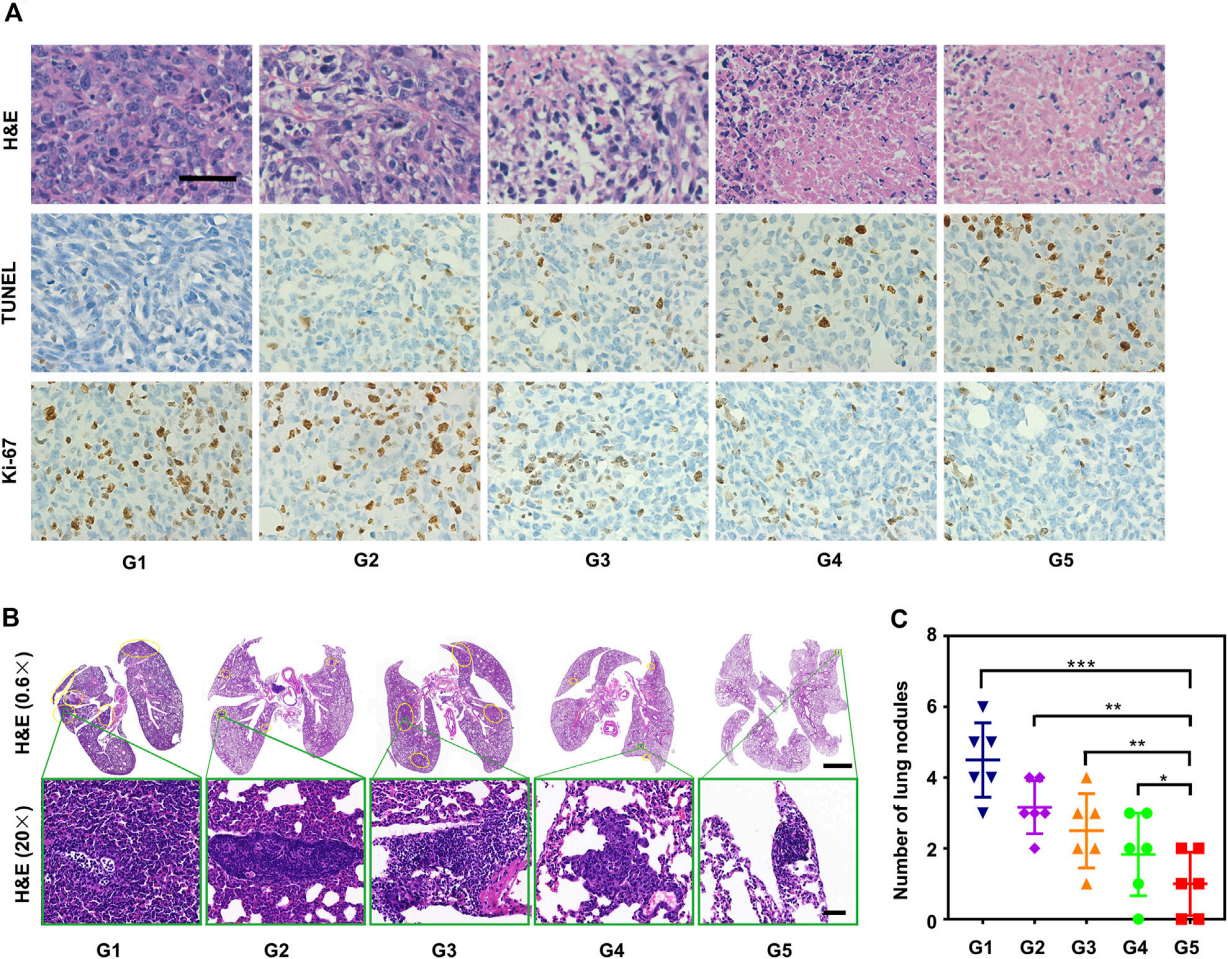
*In vivo* study of the antitumor and anti-metastasis effect of synergistic cascade strategy. **(A)** Hematoxylin and eosin (H&E) staining, Ki-67 staining, and TUNEL staining images of tumor tissue after different treatments (G1–G5) for 18 days. Scale bars represent 50 μm. **(B)** H&E staining of lung metastatic sites (yellow borders) of 4T1 tumors. Scale bars represent 2,000 μm for 0.6× and 50 μm for 20×, respectively. **(C)** The number of pulmonary metastatic nodules after various treatments (G1–G5) was calculated according to H&E staining results. The data are presented as mean ± SD (*n* = 6, **p < 0.01, ***p < 0.001).

The degree of tumor tissue damage was evaluated by immunostaining using H&E, proliferation marker Ki-67. and TUNEL essays. The results showed that the most severe morphological change and necrosis from tumor slices were observed in G5 (Figure 8A top). G5 significantly increased the apoptosis of cancer cells (Figure 8A middle) and had the lowest stained cells by Ki-67 (Figure 8A bottom). The pathological results were consistent with the tumor size and volume, indicating that G5 had the best antitumor effect.

**FIGURE 9:**
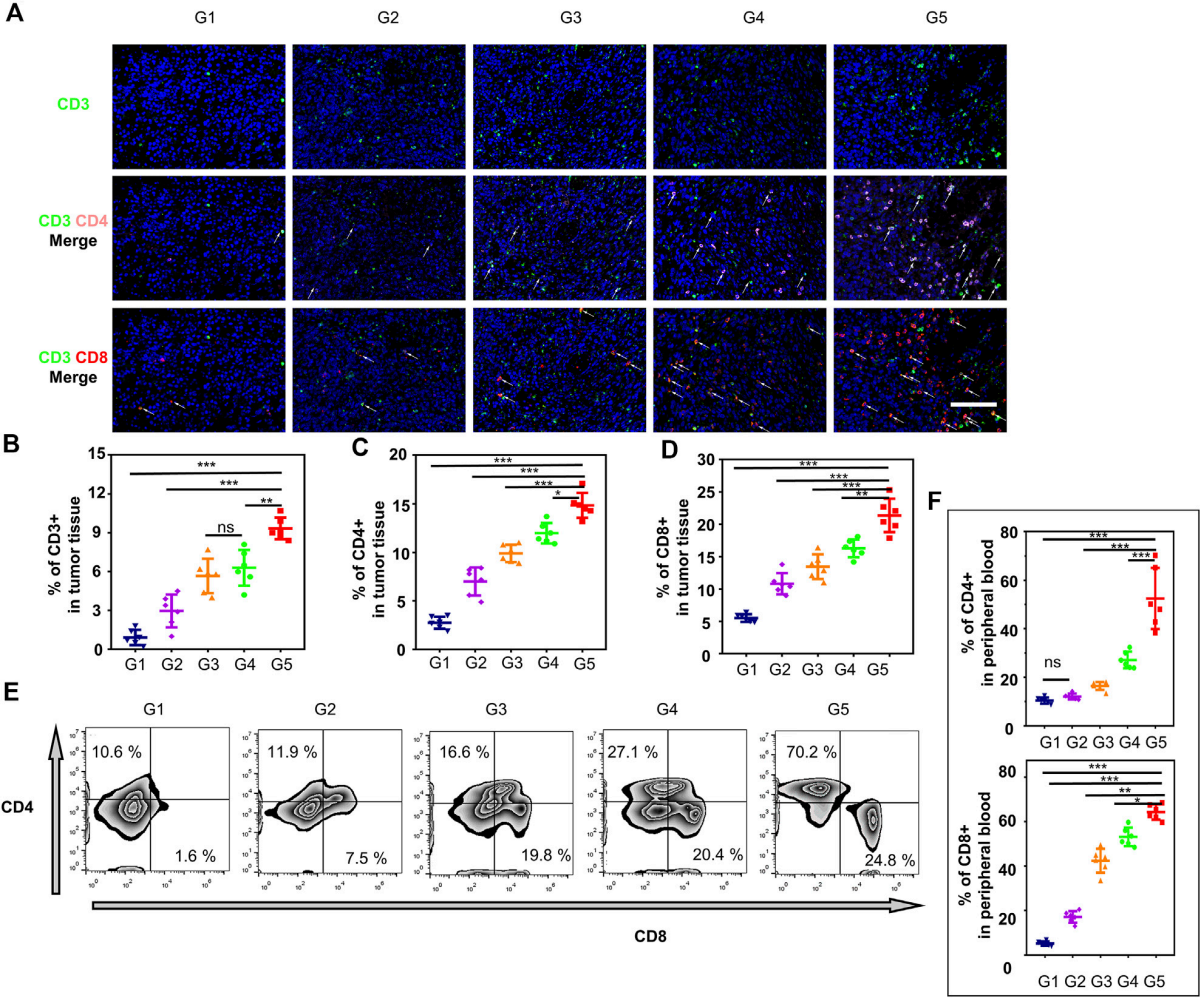
The immune effects from different treatment groups were evaluated *in-vivo*. **(A)** Representative immunofluorescence images showing tumor-infiltrating CD3^+^ CD4^+^ and CD3^+^ CD8+T cells within 4T1 tumors for different treatment groups (G1–G5). **(B–D)** percentages of CD3^+^, CD4^+^, or CD8^+^ T cells obtained from tumor tissues **(A)**. **(E)** Representative flow cytometry plots of CTLs (CD4^+^ CD8^+^) gated on CD3^+^ CTLs in peripheral blood, respectively. **(F)** Quantification (%) of CD4^+^ CD8^+^ gated on CD3^+^ CTLs. All data are presented as mean ± SD (*n* = 6, ns, not significant; *p < 0.05, **p < 0.01, ***p < 0.001).

Metastasis is a major challenge for TNBC treatment (Li et al., 2020). 4T1 breast tumor-bearing mice treated with a combination of two-step MW irradiation with HA-BNPs@Ptx exhibited a reduction in spontaneous lung metastasis (Figures 8B,C). Intratumoral immune profiling was performed in the five groups through immunofluorescence staining (Figures 9A–D). The proportions of T cells (CD3^+^) were increased in the treated group compared with G1. Among T cells, the percentages of CD4^+^ T cells and CD8^+^ T cells were significantly increased in the G5. Analysis of peripheral blood by flow cytometry was consistent with the above results (Figures 9E,F). These results suggest that sequential treatment of SCS can stimulate and recruit CD4^+^ CD8^+^ T cells in breast cancer tissues and, thus, effectively inhibit spontaneous lung metastasis.

Subsequently, cytotoxicity of HA-BNPs-Ptx was investigated *in vivo*. There was no difference in liver and kidney function (analysis of serum levels for ALT, AST, BUN, and CRE) between the PBS group and the HA-BNPs-Ptx group (Supplementary Figure S8). This indicates that the nanoparticles have good biocompatibility. The histological analysis staining of main organs tissue was done by hematoxylin and eosin (H&E) (Supplementary Figure S9). After the treatment to study the damage in mice, no tissue necrosis was observed in the main organs (heart, liver, spleen, and kidneys) for all groups, demonstrating that the SCS was safe *in vivo*.

## Discussion

In the present study, we have combined different therapeutic approaches to treat the most challenging solid cancer, TNBC. Our hypothesis is that to manage high metastasis and high recurrence of TNBC, it is necessary to have a very strong and aggressive treatment. For this, we have designed a “synergistic cascade strategy” based on modifying TME, which consists of a pretreatment of tumor and its surrounding tissue with mild hyperthermia and followed by applying SDDS. The SDDS is composed of two components: 1) intravenous injection of tumor CD44-targeted thermosensitive liposomes (TSL) preloaded with BML and Ptx, and 2) MW-induced hyperthermia to simultaneously achieve drug release and tumor ablation.

To overcome the resistance of TNBC and to improve the uptake and biodistribution of nanoparticles, the tumor was first irradiated by MW to induce a mild hyperthermia (43°C) to alter the TNBC microenvironment. This “hyperthermia-mediated EPR effect” allows to reduce the interstitial pressure of TME and increase intratumoral blood flow and thereby enhance the uptake of nanoparticles at the tumor site. Following the first MW exposure, the nanoparticles HA-BNPs@Ptx was injected into 4T1 tumor-bearing mice via tail vein. Among the hyperthermia techniques, MW heating offers highly competitive advantages: faster heat generation, less susceptibility to heat up local tissues, maneuverability, and depth of penetration in tissues (Shi et al., 2015). Although encouraging results are being collected, MW hyperthermia has its own challenges, such as inaccurate targeting and low selectivity, which lead to damage to the surrounding vital organs and tissues (Chen et al., 2017). Therefore, much effort has been devoted to improving the diffusion and the effective accumulation of the heat in the region of the whole tumor by introducing MW-sensitive agents for thermal therapy. In this study, we verified that BML can be used as MW-sensitive agents. BML is a kind of ionic liquid, which has high thermal stability, wide temperature range for the liquid state, low interfacial tension, immeasurable vapor pressure, and high ionic conductivity (Chen et al., 2020). The absorbed microwave energy is transferred to the kinetic and interionic energies of ions and stored as Joule heating energy of salt ions via the interactions between salt ions, which leads to rapid temperature rise (Sorn et al., 2019). The nanoparticles were made of thermosensitive phospholipid DPPC (Tm ≈ 41°C) and PLGA, which have been extensively used in nanomedicine formulations both for clinical and laboratory for breast cancer treatment. The second MW irradiation allowed producing a hyper heating effect (T > 50°C) due to the activation of BML and a rapid release of Ptx from nanoparticles. The increased temperature at the tumor site could directly damage tumor cells and surrounding tissues, inducing apoptosis both *in vitro* (Figure 4) and *in vivo* (Figures 7 and 8).

Hyaluronic acid is an important component of the extracellular matrix. It plays an important role in tumor microenvironment and is involved in tumor cell proliferation, invasion, immune escape, stem cell change, and drug resistance (Vasvani et al., 2020). HA is also a ligand for lymphatic vessel endothelial hyaluronan receptor-1 and hyaluronan-mediated motility receptor. HA is a biocompatible, biodegradable, and nonimmunogenic biopolymer capable of actively targeting cluster of CD44 on cell surface receptors overexpressed in many cancer cells, including TNBC (Mattheolabakis et al., 2015; Safdar et al., 2018; Wang et al., 2018; Kari et al., 2020). For all these reasons, we have selected HA as a ligand to target 4T1 tumor cells that overexpress CD44. Compared with non-targeted nanoparticles, HA-BNPs@Ptx can specifically target TNBC tumor cells both *in vitro* (Supplementary Figure S4) and *in vivo* (Figure 5). HA-BNPs@Ptx nanoparticles exhibited improved cellular uptake, probably via HA receptor-mediated endocytosis and phagocytosis (Figure 4).

In our previous work (Xu et al., 2019a), we have demonstrated that encapsulated BML in P-selectin-targeted nanoparticles could be used to boost the thermal effect of MW-generated hyperthermia and efficiently prevent tumor progression and lung metastasis in HCC tumor-bearing mice. In the present study, we further confirmed that the incorporation of BML in the liposomes can significantly enhance the thermal effect induced by MW irradiation with an increase in temperature of more than 15°C. It is worth noting that a mild MW irradiation (0.8 W cm^−2^ and 4 min) was sufficient to increase significantly the intratumoral blood flow as shown by CEUS imaging and tumor microvessel density. As a result, the retention of nanoparticles HA-BNPs@Ptx was increased by sevenfold (Figure 6).

Currently, chemotherapy remains the main option for the treatment of TNBC (Oualla et al., 2017; Núñez Abad et al., 2021). However, conventional chemotherapies employed in the treatment of TNBC suffer from issues of poor bioavailability, poor cellular uptake, resistance to drugs, and undesirable off-site toxicities (Bai et al., 2021). Abraxane, a nanoparticle formulation of “Taxol” (paclitaxel), was approved for the treatment of metastatic breast cancer, but the efficacy is limited due to the high heterogeneity and drug resistance of breast tumors (Gradishar, 2006). In the past few years, extensive research has been conducted to overcome drug resistance and improve the prognosis of TNBC (Bai et al., 2021). The combination of chemotherapeutics with SDDS, particularly with stimuli-responsive systems that are able to control drug biodistribution and release in response to specific stimuli, either exogenous (temperature, magnetic field, ultrasound, light, or electric pulses) or endogenous (changes in pH, enzyme concentration, or redox gradients) has been explored for metastatic breast cancer (Rivera-Rodriguez et al., 2018; Yang et al., 2020; Li et al., 2021). The main advantages of combination SDDS with chemotherapy are 1) the decrease in toxicity of chemotherapeutic agents (Hu et al., 2020; Thakkar et al., 2020), 2) the local delivery of anticancer agents with higher payloads and optimal distribution (Wang et al., 2021), 3) the use of nanocarriers to deliver immunomodulatory agents that can activate immune cells and modulate TME (Yang and Gao, 2017; Chen et al., 2019; Feng et al., 2020), 4) the incorporation of diagnostic agents for imaging the tumor or TME (Wang et al., 2019; Wang et al., 2020), and 5) the incorporation of active targeting molecules and specific stimuli to have synergistic cascade effects to enhance antitumor efficacy. The results obtained in this study fully demonstrate the virtue of SDDS. Finally, the use of thermal effect to pretreat the tumor and its microenvironment, which allows significantly increasing nanodrug uptake, the so-called “hyperthermia-mediated EPR effect,” is an efficient and clinically translatable approach. This approach should be further investigated and explored.

## Conclusion

In this study, we demonstrate that mild MW hyperthermia (43°–44°C) can be used to pretreat tumors to promote vasodilation of tumor vessels, improve intratumoral perfusion, and thus increase retention of nanodrugs. HA-BNPs@Ptx as SDDS is highly efficient in active targeting, MW stimulation, and induction of tumor cell apoptosis. In addition, the synergistic cascade effects by combining two-step MW irradiation and HA-BNPs@Ptx could effectively inhibit progression of triple negative breast tumor in mice. Two-step hyperthermia combined with nanomedicine showed a good synergistic cascade antitumor effect on both solid breast tumor *in situ* and lung metastasis, thus, providing a new approach for the treatment of breast cancer.

## Data Availability

The original contributions presented in the study are included in the article/Supplementary Material. Further inquiries can be directed to the corresponding authors.
