# Supplementary material for: Synergistic Cascade Strategy Based on Modifying Tumor Microenvironment for Enhanced Breast Cancer Therapy
Source: Front Pharmacol. 2021 Nov 15;12:750847. doi: 10.3389/fphar.2021.750847 (PMC8636108; doi:10.3389/fphar.2021.750847)
Supplement: Supplementary file 1 [file DataSheet1.DOC]

Supplementary Material

# Supplementary Methods

- 1. **Standard concentration curve of Ptx**

Ptx was dissolved in acetonitrile: water (V/V=8:2) at 1.25 μg mL^-1^, 2.50 μg mL^-1^, 5.00 μg mL^-1^, 10.00 μg mL^-1^, 16.66 μg mL^-1^ and 25.00 μg mL^-1^, respectively. The resulting solutions were placed in the sample cuvette and scanned at the full wavelength (190-1100 nm) with an UV-VIS-NIR spectrophotometer to obtain the maximum absorption wavelength. The maximum absorption wavelength was recorded at 229 nm and the corresponding standard concentration curve was plotted.

- 1. **Expression of CD44 on 4T1 cells**

In order to examine the expression level of CD44, 4T1 cells were seeded into 6-well plate (7.5×10^5^ cells per well). The blocked group (control group) was pre-saturated with 500 µL HA solution (5 mg/mL). No treatment was performed in the CD44 expression group. After overnight culture, 4T1 cells were stained with 10 µL anti-mouse CD44 mAb (Invitrogen, Carlsbad, CA, USA) for 30 min and 5 random fields of view were observed by an inverted fluorescence microscope (AX10, Carl Zeiss, Co., Ltd., Jena, Germany).

- 1. **Anti-tumor activity of MW hyperthermia in presence of nanoparticles**

4T1 cells were seeded into 6-well plate (7.5×10^5^ cells per well). After 24 h, 100 µL HA-BNPs without Ptx were co-incubated with 4T1 cells for another 6 h. Then, 4T1 cells were irradiated at different MW power (0.4, 0.8, 1.2 and 1.6 W cm^-2^) and for different times (1-4 min with an interval of 1 min). 1 mL Calcein-AM/PI test solution was added to each well and incubate at 37 ℃ for 30 min. 4T1 cells were imaged under the inverted fluorescence microscope. Green color denotes Calcein-AM staining (live cells) and red color indicates PI staining (dead cells), respectively.

- 1. ***In-vivo*** **Blood Chemistry Analysis**

For the blood biochemistry analysis *in-vivo*, BALB/c mice (6 mice per group) were intravenously injected with HA-BNPs@Ptx or PBS (0.5 μL g^−1^) at an equivalent dosage of body weight. After 7 days, Serum was obtained from mice to examine hepatic and renal function indicators including alanine aminotransferase (ALT), aspartate aminotransferase (AST), creatinine (CRE) and blood urea nitrogen (BUN).

# Supplementary Figures


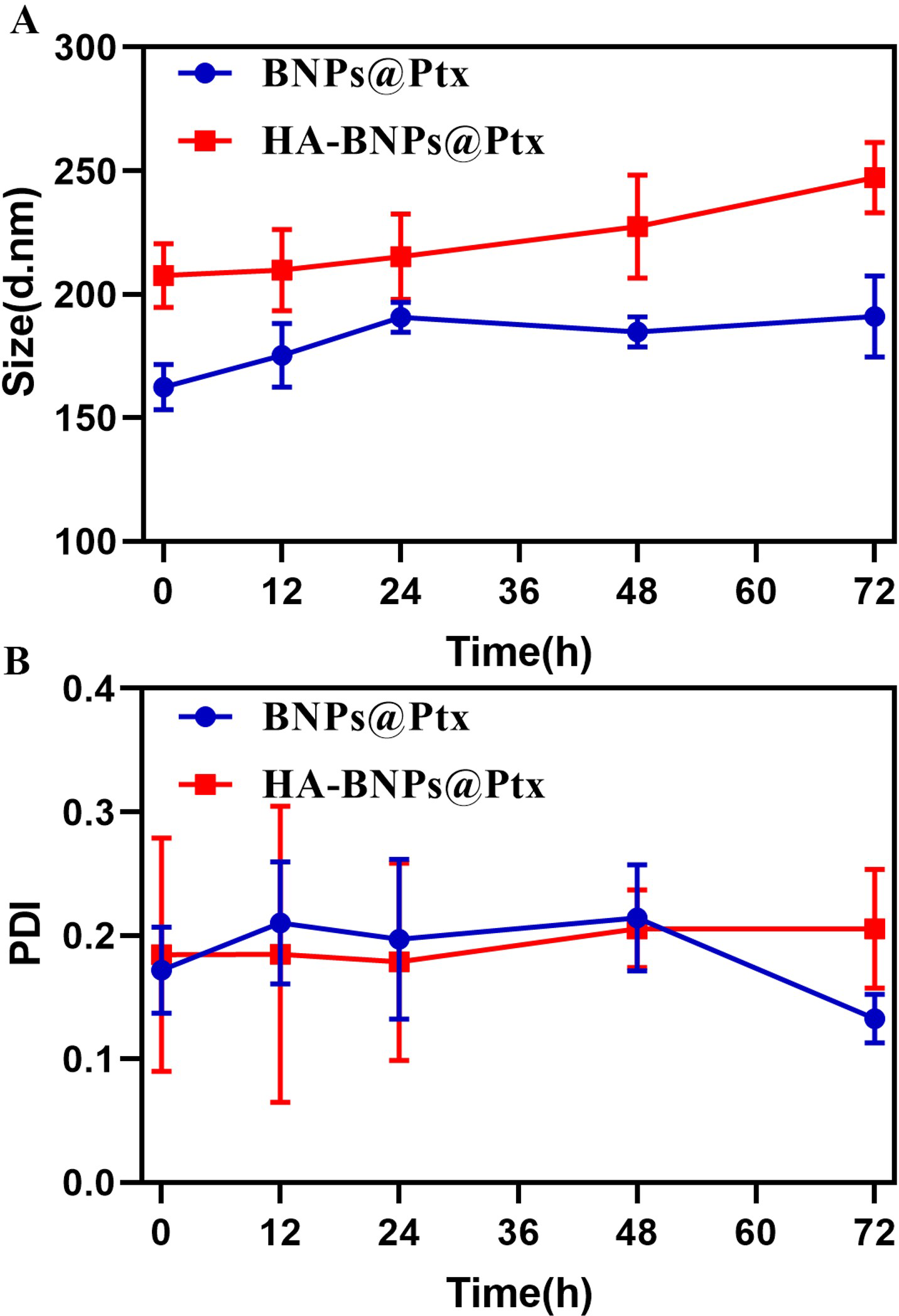


**Supplementary Figure 1.** **Stability study of nanoparticles used for SCS treatment. (A)** Changes in average diameter of BNPs@Ptx and HA-BNPs@Ptx measured by DLS in PBS at pH 7.4 and 25°C. **(B)** Changes in polydispersity index. The data were presented as mean ± SD (n=3).

**
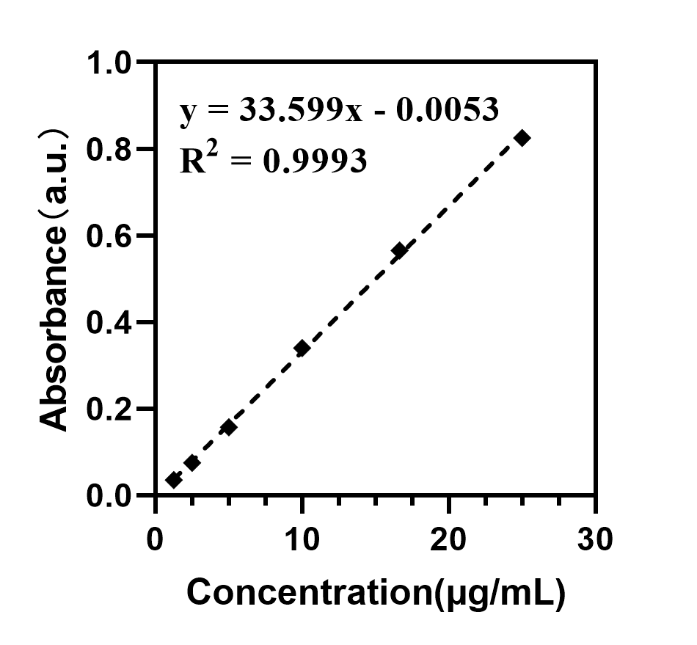
**

**Supplementary Figure 2. Standard concentration curve of Ptx.** Different concentrations of Ptx were used to build the standard curve: 1.25 μg/mL, 2.50 μg/mL, 5.00 μg/mL, 10.00 μg/mL, 16.66 μg/mL and 25.00 μg/mL.

**
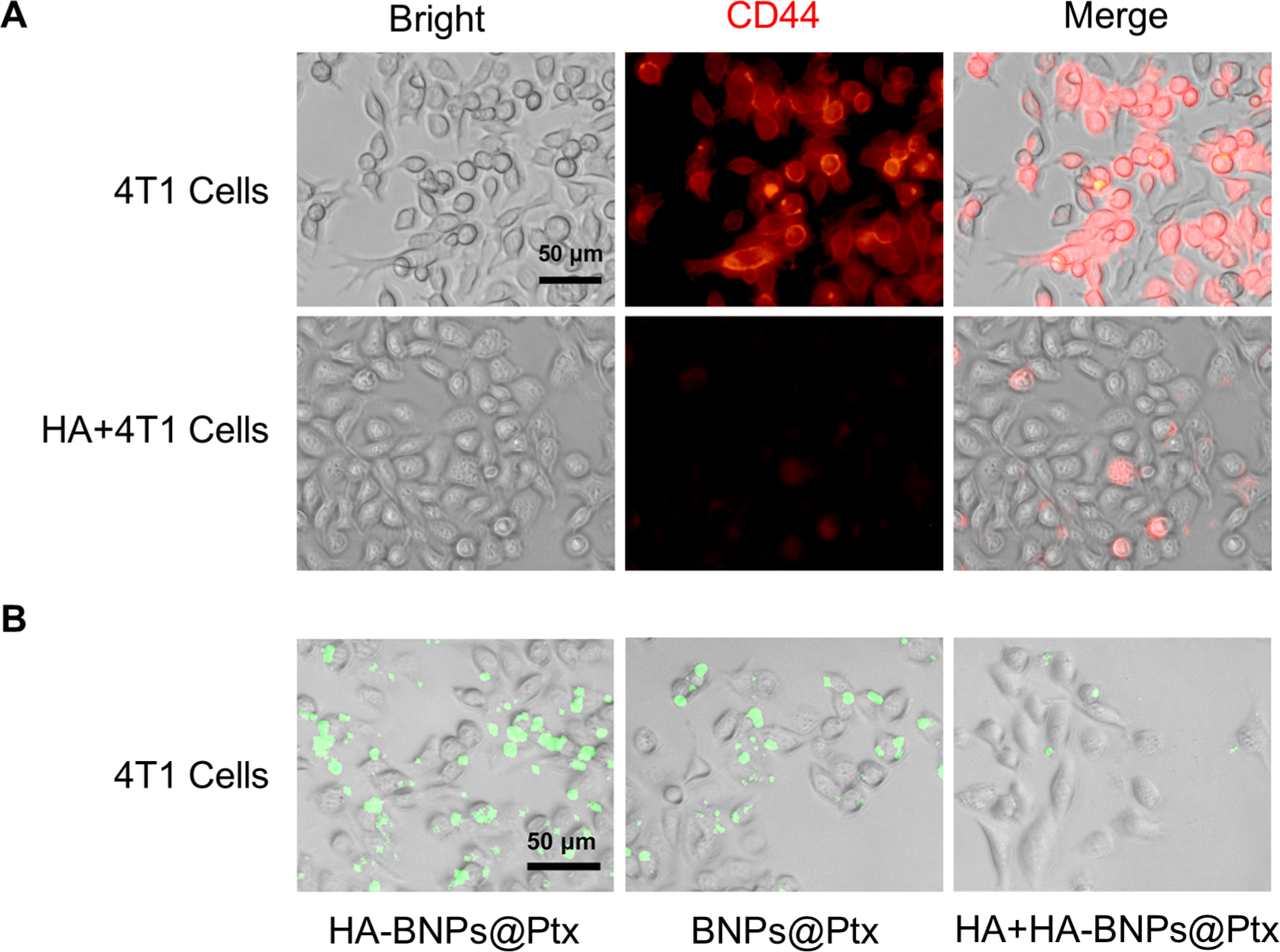
**

**Supplementary Figure 3.** **CD44 expression and targeting ability of nanoparticles to 4T1 cells. (A)** The cells expressed high level of CD44 (red), and its receptors could be blocked by pre-saturating the cells with free HA (100 μL HA at 5 mg/mL). Representative fluorescence images of 4T1 cells stained with 10 μL CD44 antibody (red fluorescence). **(B)** CD44-targeted HA-BNPs@Ptx bund significantly better than BNPs@Ptx (green) to the tumor cells. The image (right) shows that the binding could be inhibited by adding 100 μL HA (5 mg/mL) to the cells. Scale bars represent 50 μm.

**
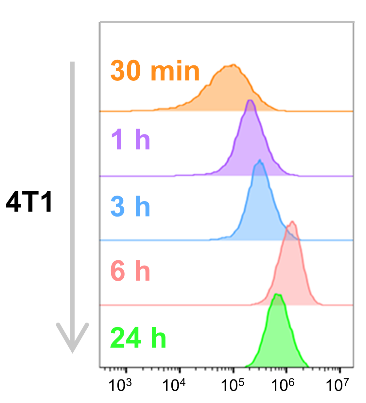
**

**Supplementary Figure 4. Flow cytometry of HA-BNPs@Ptx to 4T1 cells.** Uptake of Dil-HA-BNPs@Ptx by 4T1 cells at different time points determined with Flow cytometry.

**
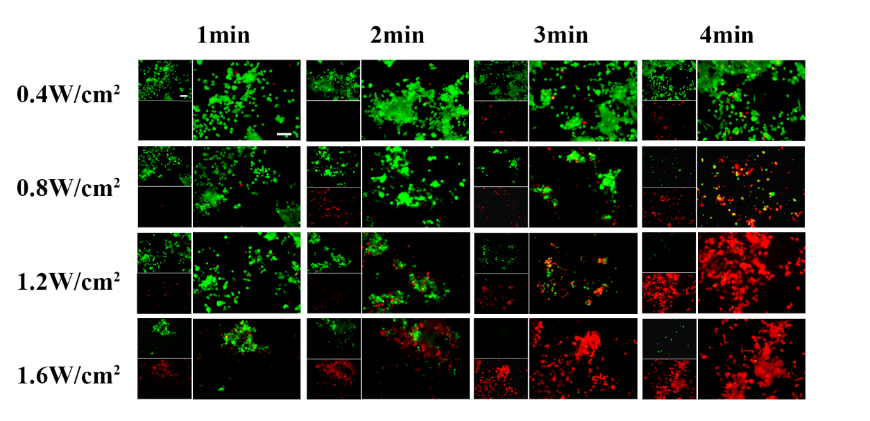
**

**Supplementary Figure 5.** ***In-vitro* thermal effect on 4T1 cells irradiated at different MW power and duration in presence of HA-BNPs.** Fluorescent images of 4T1 cells co-stained with Calcein AM (live cells, green fluorescence) and PI (dead cells, red fluorescence) upon HA-BNPs@Ptx at 24 h with MW irradiation at different power densities (0.4, 0.8, 1.2 and 1.6 W cm^-2^). Scale bars represent 50 μm.

**
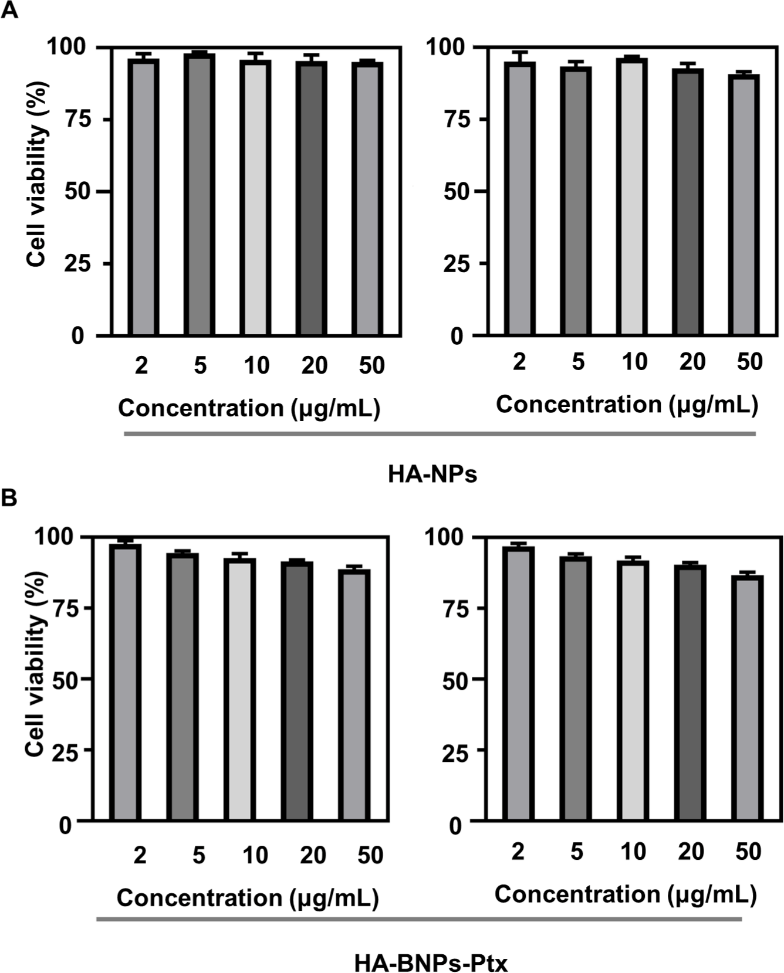
**

**Supplementary Figure 6.** ***In-vitro* cytotoxicity of HA-NPs and HA-BNPs@Ptx incubated with 4T1 cells and HUVEC. (A)** Cell viability after incubated with HA-NPs and **(B)** Cell viability after incubated with HA-BNPs@Ptx for 48 h at different concentrations (2, 5, 10, 20 and 50 mg/mL). The experiments were repeated three times independently. The data were presented as mean ± SD.


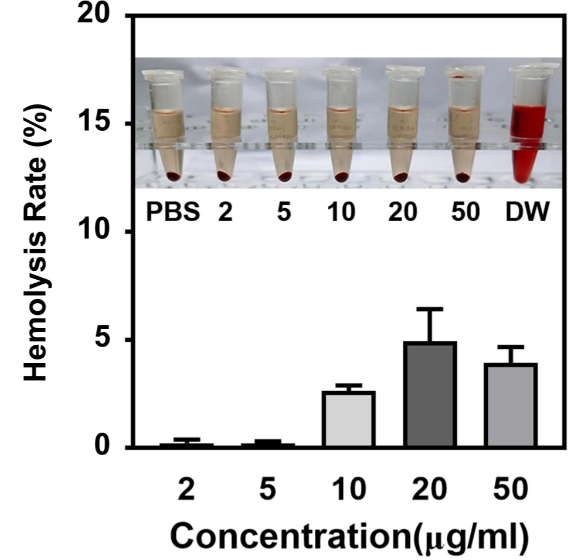


**Supplementary Figure 7. *In-vitro* hemolysis test.** Biocompatibility of picture inset showing the corresponded hemolysis images and hemolysis rate of positive control (deionized water, DW), HA-NPs@Ptx at different concentrations (2, 5, 10, 20 and 50 mg/mL), and negative control (phosphate-buffered saline, PBS), respectively. The data were presented as mean ± SD (n=6).

**
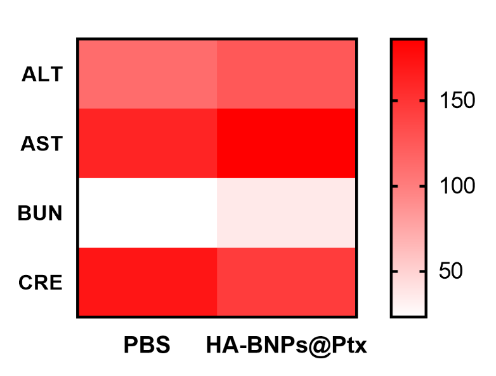
**

**Supplementary Figure 8. Liver and kidney function.** Serum levels of ALT, AST, BUN and CRE of mice 72 h after injection of PBS or HA-BNPs@Ptx. The data were presented as mean ± SD (n = 3).

**
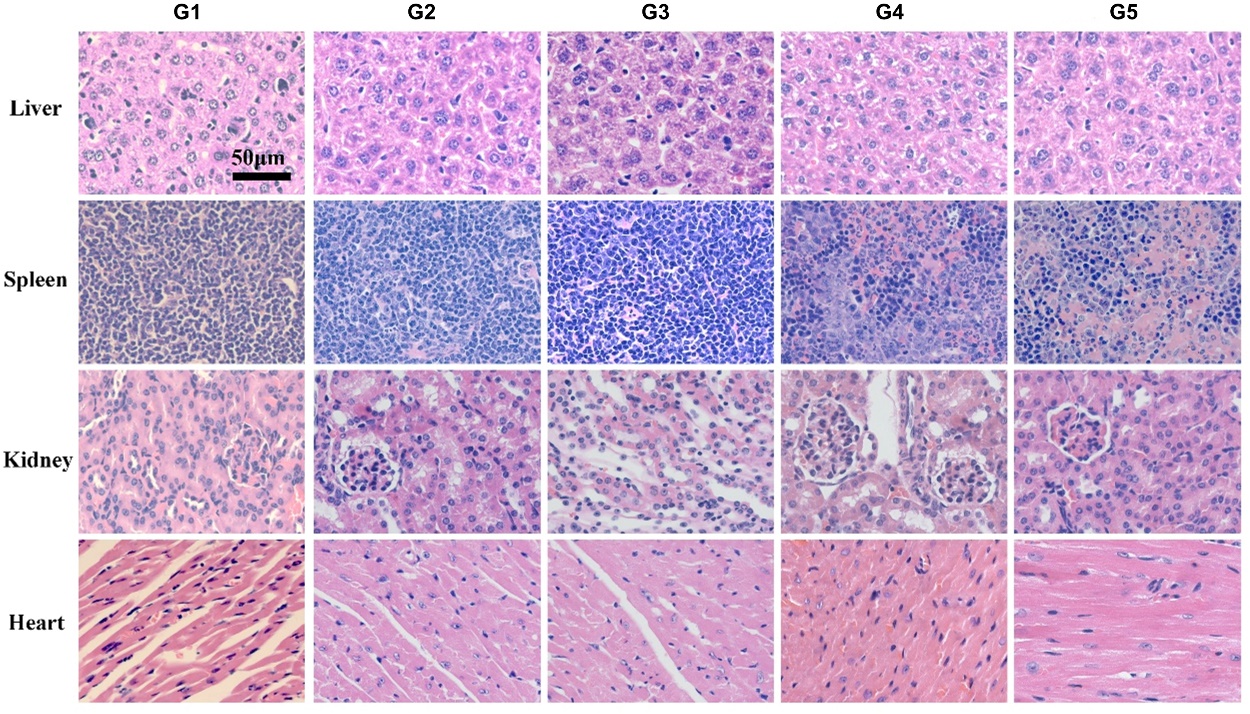
**

**Supplementary Figure 9. H&E staining of main organs.** Representative H&E staining images of liver, spleen, kidney and heart sections of the mice 18 days after the treatment with different conditions (G1-G5). Scale bars represent 50 μm (n= 6).
